# High-level nitrofurantoin resistance in a clinical isolate of *Klebsiella pneumoniae:* a comparative genomics and metabolomics analysis

**DOI:** 10.1128/msystems.00972-23

**Published:** 2023-12-11

**Authors:** Maytham Hussein, Zetao Sun, Jane Hawkey, Rafah Allobawi, Louise M. Judd, Vincenzo Carbone, Rajnikant Sharma, Varsha Thombare, Mark Baker, Gauri G. Rao, Jian Li, Kathryn E. Holt, Tony Velkov

**Affiliations:** 1 Department of Pharmacology, Monash Biomedicine Discovery Institute,Monash University, Clayton, Victoria, Australia; 2 Department of Biochemistry and Pharmacology, School of Biomedical Sciences, Faculty of Medicine, Dentistry and Health Sciences, The University of Melbourne, Parkville, Victoria, Australia; 3 Department of Infectious Diseases, Central Clinical School, Monash University, Melbourne, Victoria, Australia; 4 Doherty Applied Microbial Genomics (DAMG), 12 Peter Doherty Institute for Infection and Immunity, The University of Melbourne, Melbourne, Victoria, Australia; 5 AgResearch Limited, Grasslands Research Center, Tennent Drive, Palmerston North, New Zealand; 6 Division of Pharmacotherapy and Experimental Therapeutics, Eshelman School of Pharmacy, University of North Carolina, Chapel Hill, North Carolina, USA; 7 Discipline of Biological 17 Sciences, Priority Research Center in Reproductive Biology, Faculty of Science and IT, University of Newcastle, University Drive, Callaghan, New South Wales, Australia; 8 Department of Microbiology, Monash Biomedicine Discovery Institute, Monash University, Clayton, Victoria, Australia; 9 Department of Infection Biology, London School of Hygiene and Tropical Medicine, London, UK; Lebanese American University-Byblos Campus, Byblos, Lebanon

**Keywords:** nitrofurantoin, *Klebsiella pneumoniae*, antimicrobial resistance, genomics, metabolomics

## Abstract

Nitrofurantoin is a commonly used chemotherapeutic agent in the treatment of uncomplicated urinary tract infections caused by the problematic multidrug resistant Gram-negative pathogen *Klebsiella pneumoniae*. The present study aims to elucidate the mechanism of nitrofurantoin action and high-level resistance in *K. pneumoniae* using whole-genome sequencing (WGS), qPCR analysis, mutation structural modeling and untargeted metabolomic analysis. WGS profiling of evolved highly resistant mutants (nitrofurantoin minimum inhibitory concentrations > 256 mg/L) revealed modified expression of several genes related to membrane transport (porin *ompK36* and efflux pump regulator *oqxR*) and nitroreductase activity (*ribC* and *nfsB*, involved in nitrofurantoin reduction). Untargeted metabolomics analysis of total metabolites extracted at 1 and 4 h post-nitrofurantoin treatment revealed that exposure to the drug caused a delayed effect on the metabolome which was most pronounced after 4 h. Pathway enrichment analysis illustrated that several complex interrelated metabolic pathways related to nitrofurantoin bacterial killing (aminoacyl-tRNA biosynthesis, purine metabolism, central carbohydrate metabolism, and pantothenate and CoA biosynthesis) and the development of nitrofurantoin resistance (riboflavin metabolism) were significantly perturbed. This study highlights for the first time the key role of efflux pump regulator *oqxR* in nitrofurantoin resistance and reveals global metabolome perturbations in response to nitrofurantoin, in *K. pneumoniae*.

**IMPORTANCE:**

A quest for novel antibiotics and revitalizing older ones (such as nitrofurantoin) for treatment of difficult-to-treat Gram-negative bacterial infections has become increasingly popular. The precise antibacterial activity of nitrofurantoin is still not fully understood. Furthermore, although the prevalence of nitrofurantoin resistance remains low currently, the drug’s fast-growing consumption worldwide highlights the need to comprehend the emerging resistance mechanisms. Here, we used multidisciplinary techniques to discern the exact mechanism of nitrofurantoin action and high-level resistance in *Klebsiella pneumoniae*, a common cause of urinary tract infections for which nitrofurantoin is the recommended treatment. We found that the expression of multiple genes related to membrane transport (including active efflux and passive diffusion of drug molecules) and nitroreductase activity was modified in nitrofurantoin-resistant strains, including oqxR, the transcriptional regulator of the oqxAB efflux pump. Furthermore, complex interconnected metabolic pathways that potentially govern the nitrofurantoin-killing mechanisms (e.g., aminoacyl-tRNA biosynthesis) and nitrofurantoin resistance (riboflavin metabolism) were significantly inhibited following nitrofurantoin treatment. Our study could help inform the improvement of nitrofuran derivatives, the development of new pharmacophores, or drug combinations to support the resurgence of nitrofurantoin in the management of multidrug resistant *K. pneumouniae* infection.

## INTRODUCTION

The world is now facing a major health crisis with the emergence of multidrug resistant (MDR) Gram-negative bacteria such as the problematic opportunistic pathogen *Klebsiella pneumoniae* (1). MDR *K. pneumoniae* is a common cause of hospital-acquired infections, including urinary tract infections (UTI), pneumonia, surgical wound infections, and subsequent disseminated infections (sepsis) (2). The production of carbapenemases (KPC, NDM, OXA-48, IMP, and VIM) is the major mechanism underlying carbapenem resistance in *K. pneumoniae*, and carbapenemase-producing *K. pneumoniae* has emerged as a threatening epidemic pathogen in hospital settings worldwide, including Australia (3–5). Additionally, alterations in outer membrane permeability and upregulation of efflux systems are observed in MDR *K. pneumoniae* and contribute to resistance to multiple drug classes (3, 6, 7). The emergence of MDR *K. pneumoniae* with multiple mechanisms of resistance complicates therapy and limits treatment options to a small number of agents including polymyxins, fosfomycin, tigecycline, and nitrofurantoin (8, 9).

Nitrofurantoin is a nitrofuran prodrug that concentrates in urine and was initially introduced in 1940s for the treatment of bladder infections (10). This compound displays a broad spectrum of activity against both Gram-positive and Gram-negative bacteria, including most UTI pathogens such as *Escherichia coli*, *Klebsiella sp.*, *Enterobacter sp., Enterococcus sp*., and *Staphylococcus aureus* (11, 12). As nitrofurans concentrate in urine, they are particularly attractive as therapeutics for hospitalized patients as they risk collateral damage to the gut microbiome, which can lead to compounding issues such as *Clostridioides difficile* colitis. For this reason, they are now a recommended therapy for uncomplicated UTI in Australian hospitals and elsewhere. They also have the advantage of being orally administered, as opposed to carbapenems which require intravenous delivery. The precise mechanism(s) of the bacterial killing activity of nitrofurantoin is still uncertain. Available evidence suggests that the mode of action is related to its reduction inside bacterial cells via the flavoprotein nitrofuran reductase to form reactive metabolites, which impair ribosomal RNA, DNA, and other cellular processes such as protein synthesis (13, 14). There may also be unknown bacterial enzymes that are responsible for converting nitrofurantoin into highly reactive electrophilic intermediates (15). In *E. coli*, for example, it was initially thought that there were only two type I oxygen-insensitive nitroreductases (*nfsA* and *nfsB*) that are responsible for catalyzing the nitrofuran reduction reaction; however, more recently, an additional reductase (*ahpF*) was discovered (15).

Clinical resistance to nitrofurantoin is poorly characterized, and the European Committee on Antimicrobial Susceptibility Testing has not defined clinical breakpoints or epidemiological cut-offs for nitrofurantoin resistance in *K. pneumoniae* due to lack of data; however, they currently define *E. coli* with minimum inhibitory concentration (MIC) >64 mg/L as resistant (applies to uncomplicated UTI only) (16). Our contemporary understanding of how nitrofurantoin resistance evolves in *E.coli* centers around genetic characterizations of insertions, deletions, and missense mutations in the *nfsA* and *nfsB* genes, which knockout or impact the activity and expression levels of the respective nitroreductases (14, 17–19). The overexpression of the efflux pump operon *oqxAB* is also associated with nitrofurantoin resistance in *E. coli* and *K. pneumoniae*, as the encoded membrane transporters could recognize and efficiently expel the compound from the bacterial cell, thereby preventing its accumulation at therapeutic concentrations (20). Additionally, nitrofurantoin resistance in *E. coli* can be conferred by deletions in the *ribE* gene, which encodes lumazine synthase that is needed for riboflavin biosynthesis (21). Riboflavin is an essential precursor for the production of flavin mononucleotide (FMN), which is a pivotal cofactor for the enzymatic function of the NfsA and NfsB nitroreductases (22, 23).

In contrast to this emerging understanding of resistance mechanisms in *E. coli*, to date, there remains a dearth of understanding of the nitrofurantoin killing and resistance mechanism(s) in *K. pneumoniae*. In the present study, we employed a combination of genetic analysis (whole-genome sequencing, qPCR analysis, and mutational mapping on molecular models) in combination with untargeted metabolomics to elucidate the mechanism(s) of nitrofurantoin action and resistance in a clinical *K. pneumoniae* isolate cultured from a hospital patient with UTI and experimentally evolved resistant mutants. The presented findings highlight that high-level nitrofurantoin resistance in *K. pneumoniae* is governed primarily by perturbations across several complex interrelated metabolic pathways, and resistance is associated with mechanisms that decrease drug concentration inside the cell by increasing efflux and, potentially also, by reducing porin-mediated entry and limiting the reductive activation of nitrofurantoin.

## RESULTS AND DISCUSSION

### Mutations arising in *K. pneumoniae* INF348 strains during nitrofurantoin exposure

**Fig 1:**
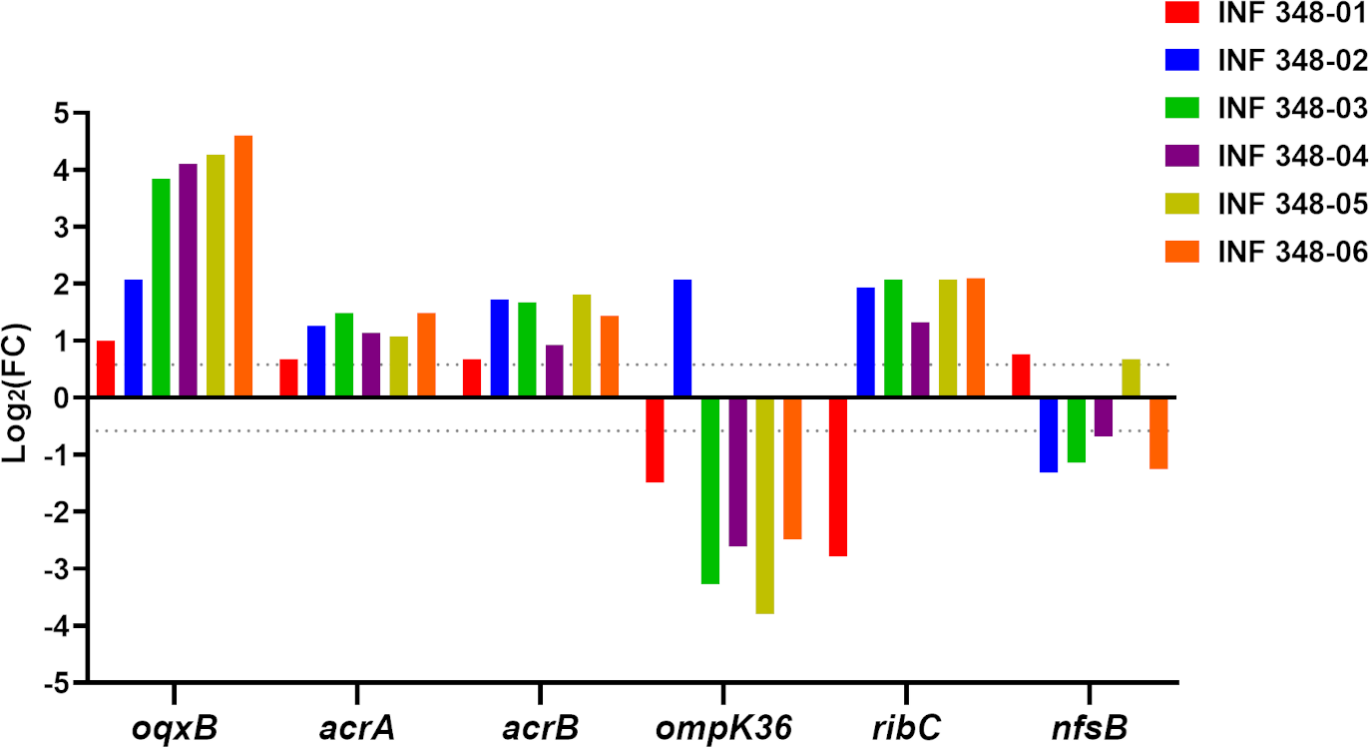
Bar graphs show the differential expression of *oqxB*, *acrA*, *acrB*, *ompC*, *ribC*, and *nfsB* genes in six nitrofurantoin-resistant *K. pneumouniae* isolates compared with the wild-type isolates *K. pneumouniae* INF348. The data for each gene were derived from the average of three biological replicates, using the delta-delta Ct method. The grid line in the chart represents the log_2_FC cut-off of ≥0.59 or ≤−0.59.

The nitrofurantoin-susceptible clinical isolate *K. pneumoniae* INF348 (MIC 32 mg/L) was passaged in increasing nitrofurantoin concentrations, and as a result, we obtained six related mutants that were stable in the presence of high levels of nitrofurantoin (MICs ≥ 256 mg/L). These high-level resistant mutants were designated as INF348-01, INF348-02, INF348-03, INF348-04, INF348-05, and INF348-06. Comparative mapping of the genome sequences of each resistant mutant vs the wild-type parent strain *K. pneumoniae* INF348 revealed three insertion sequence (IS)-mediated disruptions and seven non-synonymous single-base substitutions, affecting four genes (*oqxR*, *ompK36*, *ribC*, and *nfsB*; see Table 1). Changes in gene expression, in the resistant mutants vs wildtype progenitor, were measured via qPCR for *oqxB*, *acrAB*, *ompK36*, *ribC*, and *nfsB* (Fig. 1). All of the nitrofurantoin-resistant mutants overexpressed *oqxB* and *acrAB* [log_2_ fold change (FC) ≥0.59, corresponding to a fold change of approximately 1.5 in linear scale, relative to the wildtype progenitor], and downregulation of *ompK36* and *ribC* was also observed in selected mutants (Fig. 1). Two resistant mutants harbored substitution mutations in *nfsB*, resulting in amino acid substitutions at positions 46 or 198 of the NfsB protein sequence (Table 1).

**TABLE 1:** Mutations identified in resistant mutants derived from *K. pneumoniae* INF348^*a*^

Mutant strain (MIC)	Mutations identified				Mutation count
	*oqxR*	*ompK36*	*ribC*	*nfsB*	
INF348-01 (256 mg/L)			IS*1 × 2* (37 bp upstream)		1
INF348-02 (512 mg/L)		Q286*		W46C	2
INF348-03 (512 mg/L)		K4*	R75H		2
INF348-04 (512 mg/L)		K4*		D198Y	2
INF348-05 (512 mg/L)	ISKpn26 (coding region)	K4*			2
INF348-06 (512 mg/L)	N38K	IS1 *× 2* (47 bp upstream)			2

^*a*^ Note: “*” means stop codon.

The *oqxR* gene was altered in two resistant mutants: one via ISKpn26 insertion and one via non-synonymous mutation (N38K). OqxR is a transcriptional repressor of the *oqxAB* efflux pump, such that disruptions in *oqxR* are expected to result in de-repression of *oqxAB* and increased efflux (24). The *oqx* operon is a core locus present in most *K. pneumoniae* genomes (25). Overexpression of *oqxAB* has been previously reported to be involved in nitrofurantoin resistance in *K. pneumoniae* and has also been noted as a determinant of resistance to nitrofurantoin and other antimicrobials in *E. coli* and *K. pneumoniae* (25–27). Here, while the elevation of *oqxB* expression was observed in all high-level nitrofurantoin-resistant mutants, it is noteworthy to underscore that those with *oqxR* mutations showed the greatest overexpression (log_2_FC > 4.2), which aligns with the removal of *oqxR*-mediated repression (Fig. 1) (24). However, it remains imperative to acknowledge that other strains, such as INF-348–04, also displayed a notable 4.0-log_2_FC increase in *oqxR* expression. This suggests a wider spectrum of heightened expression occurrences, which might necessitate a comprehensive assessment of the intricate interplay of determinants influencing *oqxR* expression in strains tested.

The *ompK36* gene encodes an outer membrane porin (homologous to *E. coli ompC*) (28, 29) and was disrupted in five of the six resistant mutants (Table 1). Disruption of *ompC* homologs has been associated with reduced susceptibility to carbapenems and cephalosporins in *K. pneumoniae* (30) and other Enterobacteriaceae (31, 32) and to nitrofurantoin in *E. coli* (33); however, we could not identify any prior reports of *ompK36*/*ompC* mutations related to nitrofurantoin resistance in *K. pneumoniae*. INF348-06 harbored an IS*1 × 2* insertion 47 bp upstream of *ompK36*, which is within the promoter region defined for *E. coli ompC* (34). Consistent with this, INF348-06 showed significantly reduced *ompK36* expression (log_2_FC FC = −2.49). It has previously been reported that an IS*5* insertion 37 bp upstream of *ompK36* reduced its expression in *K. pneumoniae* ST258, resulting in resistance to carbapenems but not colistin (nitrofurantoin was not tested) (35). Notably, *ompK36* expression was also reduced in four other isolates, including three (INF348-03, INF348-04, and INF348-05) that shared a common substitution resulting in premature termination of translation of the OmpK36 protein after just four amino acids (Table 1; Fig. 1).

The *nfsB* gene encodes a nitroreductase that directly interacts with nitrofurantoin, as outlined above. Here, we detected substitution mutations in *nfsB* in two resistant mutants (INF-348 02 and INF-348 04), resulting in amino acid substitutions at positions 46 or 198 of the NfsB protein sequence (Table 1). Both of these isolates, plus two others with wildtype *nfsB*, showed evidence of downregulation of *nfsB* expression (log_2_FC ≥ −0.68); the other two isolates showed minor upregulation relative to the wildtype progenitor isolate (log_2_FC ≥ 0.68). Modification or loss of NfsB is a well-known mechanism of nitrofurantoin resistance in *E. coli*, leading to inactivated or poor catalytic function, which in turn cripples the cells’ ability to reduce nitrofurantoin into its toxic intermediate compounds (22).

The *ribC* (riboflavin synthase) and *ribB* (3,4-dihydroxy 2-butanone 4-phosphate synthase) genes are involved in riboflavin biosynthesis, a universal precursor of FMN and flavin adenine dinucleotide (FAD) (36). FMN is an essential cofactor for bacterial nitroreductases such as NfsA and NfsB that are involved in the reduction of nitrofurantoin into active antibacterial metabolites (37). Here, one resistant mutant acquired an amino acid substitution in RibC at codon 75, and another (INF348-01) had an IS1 *×* 2 insertion 37 bp upstream of *ribC*. This insertion likely disrupts the *ribC* promoter, as the gene was significantly underexpressed in INF348-01 (log_2_FC ≥ −2.79).

The resistant mutant INF348-01 acquired only one mutation (*ribC* disruption) to which its increase in MIC (to 256 mg/L) can be attributed. The other five resistant mutants harbored two mutations each, making it difficult to draw conclusions about the effects of individual mutations; however, these isolates all had an even higher MIC (512 mg/L), and all mutations were in genes with plausible relevance to nitrofurantoin resistance as outlined above. Taken together, these findings provide supportive evidence that multiple genetic mutations are associated with nitrofurantoin resistance in *K. pneumoniae,* spanning across two key resistance tiers: (i) reducing the amount of drug accumulating inside the bacterial cell by repression of drug influx (disruption of *ompK36*) and/or enhancement of drug efflux (de-repression of the *oqxAB* efflux pump via *oqxR* disruption); and (ii) crippling the enzymatic activity of the activating nitroreductases and thus reducing the accumulation of reactive metabolites, either by direct modification of *nfsB* or via inhibition of FMN cofactor synthesis.

### Mapping the mutations onto the modeled structure of *K. pneumouniae nfsB* nitroreductase

**Fig 2:**
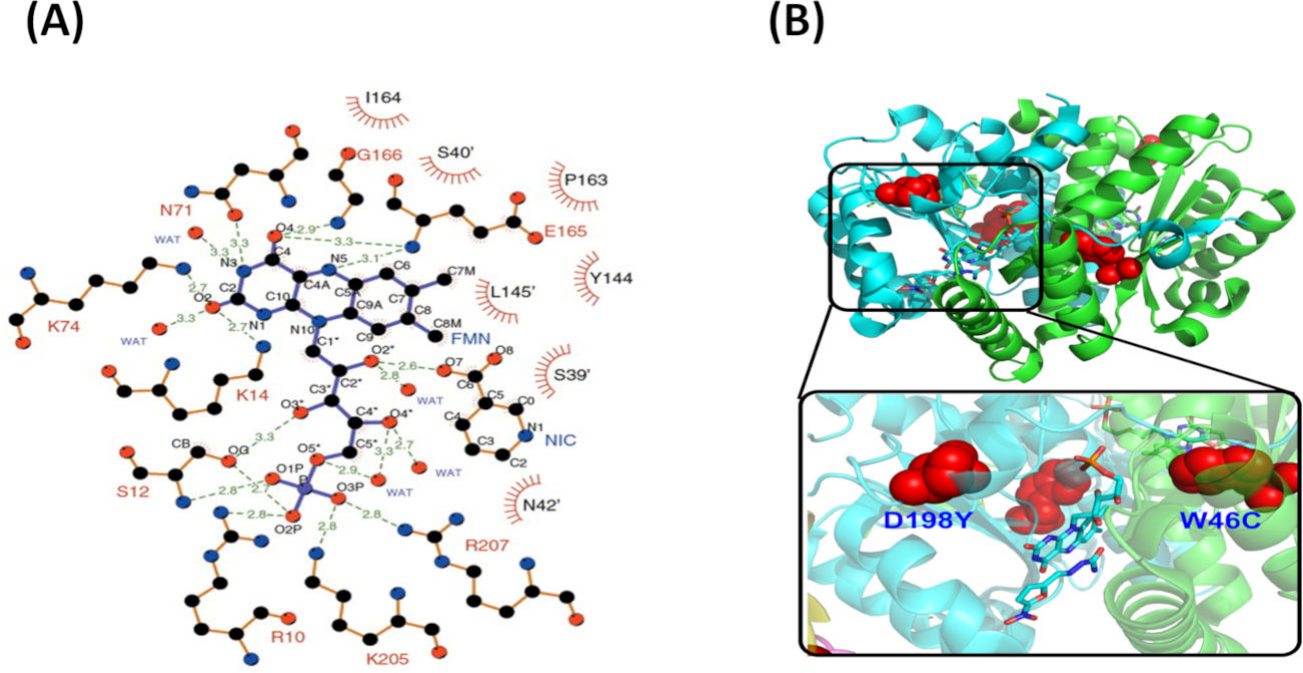
(**A**) Interaction between amino acid residues and FMN plotted by LigPlot+: amino acid residues identified that are essential for stabilizing FMN of *K. pneumouniae* NfsB and (**B**) the mapping of critical mutations D198Y and W46C onto the dimeric structure of the enzyme utilizing a space-filling representation for the residues. NIC = nicotinic acid.

A structural model of NfsB was constructed in Molsoft using the *E. coli* crystal structure of NfsB nitroreductase 1YKI bound with the nitrofuran antibiotic nitrofurazone and FMN as the modeling template (38). The modeled *K. pneumouniae* structure had 84% sequence identity with the *E. coli* protein and near-identical antibiotic and cofactor binding domains, with the exception of His11 and Asn67 (Tyr11 and Thr67 for *K. pneumoniae*; Fig. 2A). In order to help understand the functional significance of the *nfsB* mutations, INF 348–02 and INF 348–04 mutational analysis was also carried in Molsoft to quantify the potential change in protein stability (Fig. 2B ; Table 2) (39, 40). The substitution mutations were localized proximal to the NfsB active site. Importantly, the mutations are settled on an interface region of the pocket where FMN cofactor and nitrofuran binding occurs. Thus, it is likely that these mutations attenuate the ability of nitroreductase to bind FMN and nitrofuran drugs. The modeled structure indicates there are several indispensable amino acids responsible for complexation with FMN that were proximal to and thus likely impacted by the aforementioned active site mutations (Fig. 2A). These included Arg207 and Lys205 that form hydrogen bonds with phosphate of FMN and ensure that the homodimer is in the active conformation. Leu145 and Tyr144 form hydrophobic interactions with the benzenoid portion of FMN. Glu165 and Gly166 contain amide groups that interact with N5 and O4 in the isoalloxazine ring system of FMN. Furthermore, residues Arg10, Ser12, and Ser39 are involved in interactions with ribityl chain of FMN. Residues Lys14, Asn72, Glu73, Arg74, and Lys75 interact with O2, N3, and O4 in pyrimidine of FMN (Fig. 2A).

**TABLE 2:** Molsoft computation of the Gibbs free energy change in protein stability of a single-point mutation

Enzyme	Chain	Residue	Wild-type	Mutant	ddG
**^*a*^**nfsB	a	198	Asp	Tyr	1.8912
nfsB	b	198	Asp	Tyr	2.0419
nfsB	a	46	Trp	Cys	5.7388
nfsB	b	46	Trp	Cys	5.6386

^*a*^ The free energy change is displayed as ddG (kcal/mol). nfsB = nitroreductase enzyme.

The comparative sequence and structural alignments show that the aforementioned key amino acids in NfsB are identical between *E. coli* and the *K. pneumoniae* isolates in our study, indicating that these conserved amino acids are important for cofactor binding (Fig S1) (40). All NfsB mutations identified in this study were situated in positions in or next to an α-helix or β-sheet; therefore, they could potentially perturb these secondary structures. In INF 348–04, the mutation D198Y is next to an α-helix composed of amino acids fraction of conserved native contacts (Fig S1; Fig. 2B). When Asp198 is replaced by the bulkier Tyr sidechain, we suspect that the mutation perturbs the formation of the α-helix immediately preceding the mutation, in turn solvent exposing the hydrophobic residue of Phe199. This mutation in part could also affect the loop region (residues 10–14) immediately adjacent to the residue, in turn affecting a number of hydrogen bonds with FMN. Both scenarios could also affect the distance of Arg207 and Lys205 to FMN and render them unable to form hydrogen bonds.

In addition to Arg207 and Lys205, other amino acids from positions 203−209, e.g., Leu or Pro could not offer an amine or hydroxyl group to form hydrogen bonds with FMN. The Trp46Cys mutation in INF 348–02 immediately precedes a β-sheet comprising a series of hydrophobic residues and partially π-stacks with Gln44 which forms hydrogen bond contacts with the amides of the adjacent monomer and residue Leu208 (Fig S1; Fig. 2B). In the β-sheet, amino acids are stabilized by mutual hydrogen bonds and interaction of their side chains, and in this case, it is a water excluding hydrophobic patch. When position 46 is harbored by Trp, the benzenoid portion in its side chain helps to stabilize this patch and stacking arrangement with Gln44 enabling hydrogen bonds to be maintained. Replacement with a Cys could affect the overall stability of the homodimer and the positioning of the FMN-binding loop residues of Leu208 and Arg207. Overall, the stability of the dimeric enzyme is predicted to be readily affected with an increase in delta delta G (ddG) for each modeled mutant (Table 2), in particular, W46C (with an average ddG of 5.69 kcal/mol), indicating that dimerization is no longer spontaneous. In conclusion, the Asp198Tyr and W46C mutations in NfsB are likely to negatively influence the binding to FMN and in particular the overall fold and stability of the enzyme itself.

### Metabolite profiling of wild-type *K. pneumoniae* INF348 following nitrofurantoin treatment

To explore the impact of nitrofurantoin exposure on the metabolism of *K. pneumoniae*, we performed metabolite profiling on INF348 before and after exposure to 48 mg/L of nitrofurantoin, using IDEOM (see Materials and Methods). A total of 1,007 putatively identified metabolites were obtained at 1 and 4 h post exposure, which included 73 metabolites involved in carbohydrate metabolism, 174 in amino acid metabolism; 326 in peptide metabolism, 49 in nucleotide metabolism, and 110 in lipid metabolism. Univariate analysis [*t* tests, False Discovery Rate adjusted *p*-value ≤0.05; log_2_-fold change (FC) ≥0.59, corresponding approximately to a 1.5-fold change in linear scale] was used to compare post-exposure levels to pre-exposure levels to identify which metabolites were significantly altered in response to nitrofurantoin treatment. The reproducibility of all sample groups was within acceptable limits at both experimental time points, wherein the median relative SD across was 19%–26% for the untreated control groups and 18%–26% for the treated groups, which is consistent with some baseline variability in the dynamics of ordinary bacterial metabolism irrespective of nitrofurantoin treatment (Table S1). Principal component analysis illustrated that, for each post-exposure timepoint analyzed, the first principal component (responsible for >70% of the variation in each comparison) separated the treated and untreated samples. Importantly, a gap between the control and treated samples was seen at 1 and 4 h, which reflected the significant variance in the abundance between the two groups (Fig S2; Table S2). Heatmaps of metabolite profiles indicate that nitrofurantoin induced marked changes in the metabolite (mainly reduced) intensities compared to the control group; particularly at 4 h (Fig S3).

Nitrofurantoin treatment significantly perturbed the bacterial metabolome with 433 (1 h) and 518 (4 h) significantly altered metabolites relative to the untreated control group (Fig S4A ). The classification of the significantly perturbed metabolites indicated that peptides, amino acids, lipids, and carbohydrates were largely perturbed compared to other metabolites classes; and the abundance of almost all of the impacted metabolite classes declined at both 1 and 4 h, except for an elevation of lipid abundance (Fig S4B). The Venn diagram data plot showed that there were 89 and 174 unique significantly impacted metabolites of in response to nitrofurantoin treatment at 1 and 4 h, respectively (Fig S4C). Notably, 344 significant metabolites were in common across the 1 and 4 h time points (Fig S4C).

### Pathways analysis of *K. pneumoniae* the wild-type INF348 induced by nitrofurantoin treatment at 1 and 4 h

The most significantly perturbed pathways in the *K. pneumoniae* INF348 wild-type metabolome following nitrofurantoin treatment at both time points (1 and 4 h post-exposure) were aminoacyl-tRNA biosynthesis, purine, riboflavin, nicotinate and nicotinamide metabolism, pantothenate and CoA biosynthesis, and tricarboxylic acid cycle (TCA cycle) (Fig S5). In the interest of brevity, central carbohydrate metabolism (TCA and glycolysis), pantothenate, and CoA biosynthesis dysregulations are discussed in detail in the supplementary document.

#### Aminoacyl-tRNA biosynthesis

**Fig 3:**
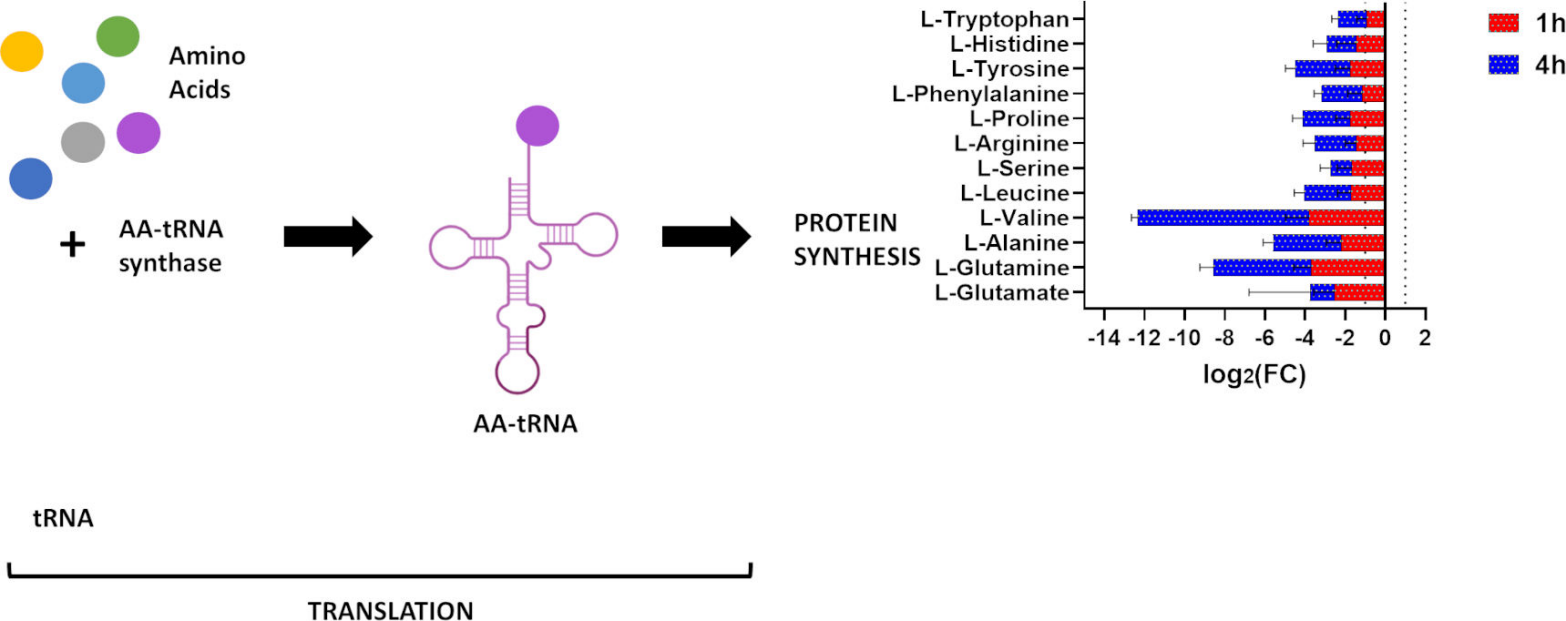
Schematic diagram shows the aminoacyl-tRNA biosynthesis and fold changes of significantly impacted metabolites *K. pneumoniae* INF348 following nitrofurantoin treatment at 1 and 4 h (log_2_FC ≥ 1.0, *P* ≤ 0.05). Data are mean ± SD for four biological replicates. The grid line in the chart represents the log_2_FC cut-off of ≥1 or ≤ −1.

Nitrofurantoin induced a significant reduction in the abundance of 13 essential amino acids involved in aminoacyl-tRNA biosynthesis at both time points (1 and 4 h), albeit the impact was greatest in intensity at 4 h (Fig. 3). The perturbed metabolites included L-glutamine, L-glutamate, L-tyrosine, L-valine, L-alanine, L-proline, L-leucine, L-tryptophan, L-arginine, L-phenylalanine, L-histidine, L-threonine, and L-serine (log_2_FC ≤ −1.0, *P* ≤ 0.05; Fig. 3).

#### Purine metabolism

**Fig 4:**
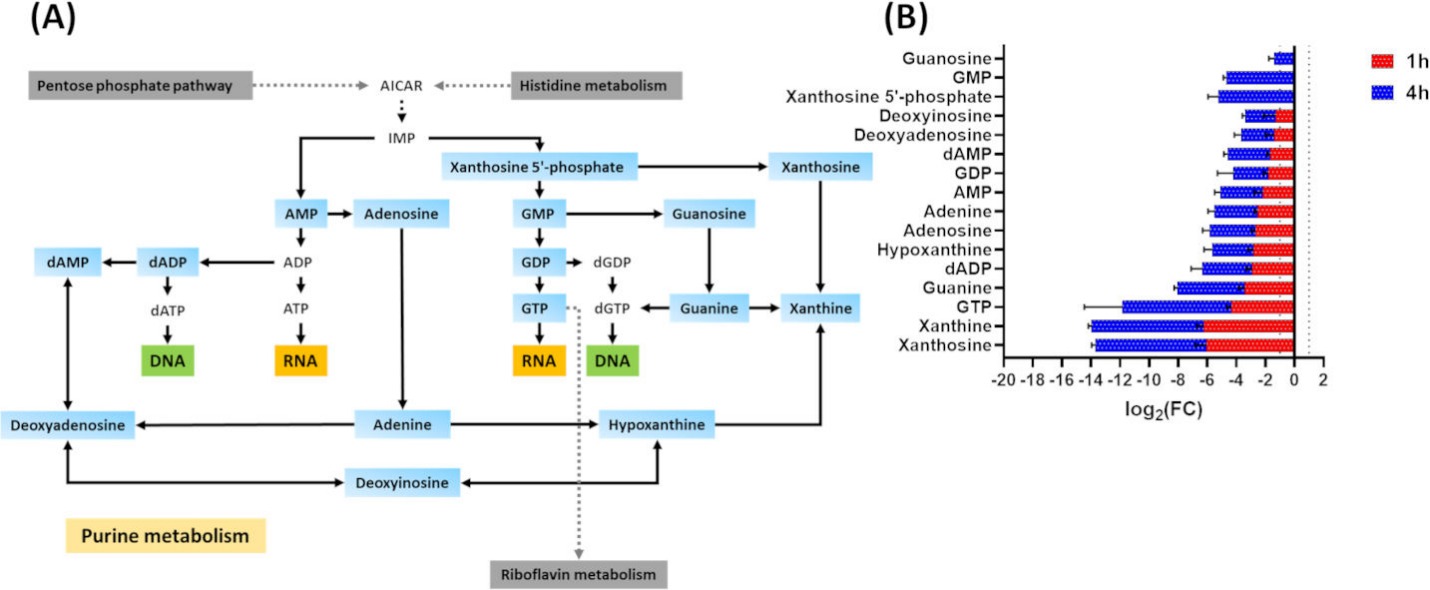
(**A**) Schematic diagram depicting the significantly impacted purine metabolism of *K. pneumoniae* INF348 following nitrofurantoin treatment at 4 h. (**B**) Bar charts for the significantly impacted intermediates from purine metabolism of *K. pneumoniae* INF348 treated with nitrofurantoin at 1 and 4 h. (log_2_FC ≥ 1.0, *P* ≤ 0.05). Data are mean ± SD for four biological replicates. Blue rectangles: significantly decreased metabolites; red rectangles: significantly increased metabolites. The grid line in the chart represents the log_2_FC cut-off of ≥1 or ≤ −1.

Nitrofurantoin treatment induced significant perturbations in the abundance of several metabolites involved in purine metabolism at 1 h and to a greater extent at 4 h (Fig. 4A and B). The bacterial response to nitrofurantoin treatment at 1 h manifested as a decrease in the levels of 13 key purine intermediates, namely xanthosine, xanthine, GTP, guanine, dADP, hypoxanthine, adenosine, adenine, AMP, GDP, dAMP, deoxyadenosine, and deoxyinosine (log_2_FC ≤ −1.0, *P* ≤ 0.05; Fig. 4B). Similarly, the abundance of 16 purines markedly declined at 4 h post nitrofurantoin exposure, namely xanthosine, xanthine, GTP, guanine, dADP, hypoxanthine, adenosine, adenine, AMP, GDP, dAMP, deoxyadenosine, deoxyinosine, xanthosine 5'-phosphate, guanosine, and Guanosine monophosphate (GMP) (log_2_FC ≤ −1.0, *P* ≤ 0.05; Fig. 4A and B).

The observed perturbations of aminoacyl-tRNA biosynthesis and purine metabolism are in line with the primary mode of bacterial killing instigated by the unstable nitrofurantoin metabolites, which involves disruption of bacterial DNA, RNA, and protein synthesis (14, 41–43).

#### Nicotinate and nicotinamide metabolism

**Fig 5:**
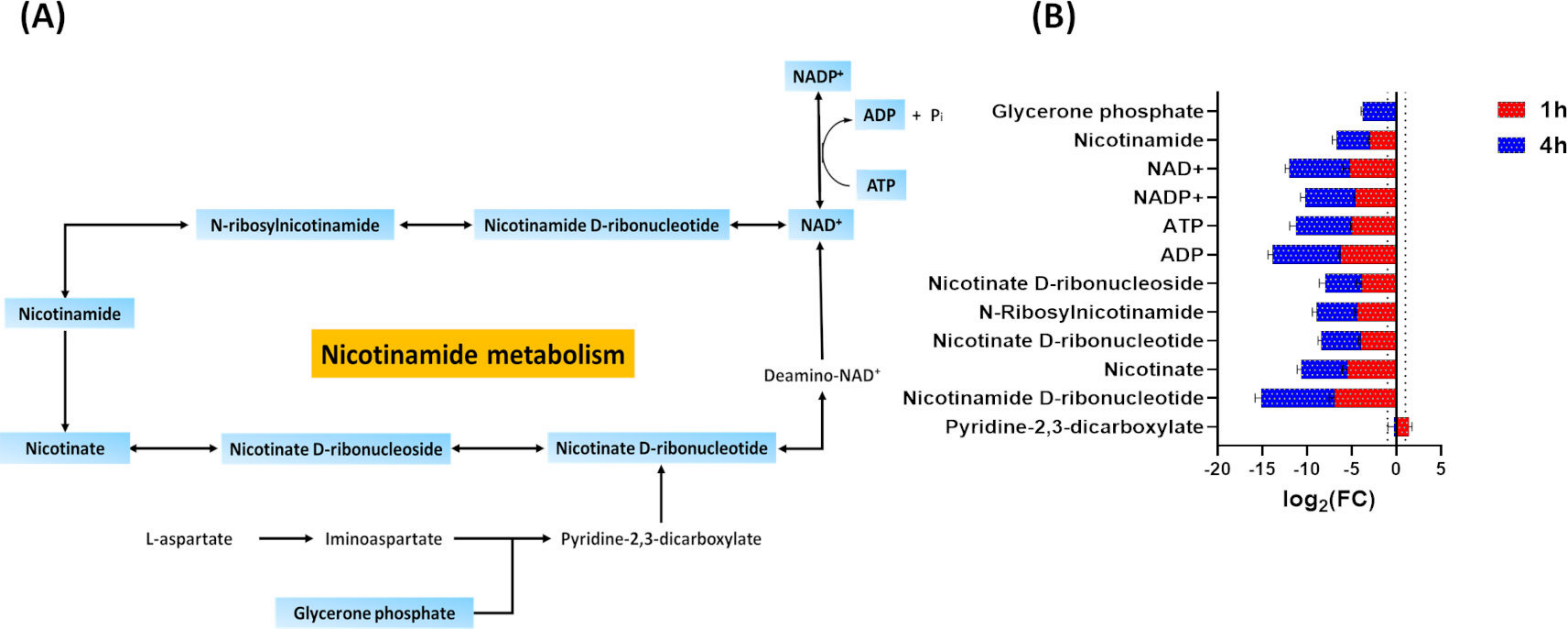
(**A**) Diagram of significantly influenced nicotinate and nicotinamide metabolism of *K. pneumoniae* INF348 after nitrofurantoin treatment at 4 h. (**B**) Fold changes for the significantly impacted metabolites from nicotinate and nicotinamide metabolism in *K. pneumoniae* INF348 treated with nitrofurantoin at 1 and 4 h (log_2_FC ≥ 1.0, *P* ≤ 0.05). Data are mean ± SD for four biological replicates. Blue rectangles: significantly decreased metabolites; red rectangles: significantly increased metabolites. The grid line in the chart represents the log_2_FC cut-off of ≥1 or ≤ −1.

The nicotinamide adenine dinucleotides (NAD^+^ and NADP^+^) are critical in generating the main components of coenzymes involved in redox reactions and for enabling electron transport (44). Nitrofurantoin treatment induced significant perturbations in the abundance of fundamental components of nicotinate and nicotinamide metabolism at both time points, particularly at 4 h (Fig. 5A and B). The levels of NAD^+^, NADP^+^, ATP, ADP, nicotinate, nicotinate D-ribonucleotide, nicotinamide D-ribonucleotide, nicotinamide, *N*-ribosylnicotinamide, and nicotinate D-ribonucleoside underwent a significant decline following nitrofurantoin treatment at 1 h (log_2_FC ≤ −1.0, *P* ≤ 0.05; Fig. 5B). A more pronounced reduction in the abundance of the same intermediates (log_2_FC ≤ −3.0, *P* ≤ 0.05) and, additionally, glycerone phosphate (log_2_FC = −10.0) was seen following nitrofurantoin exposure at 4 h (Fig. 5A and B). Interestingly, the abundance of only one intermediate [pyridine-2,3-dicarboxylate *syn*. quinolinic acid (log_2_FC = 1.37)] significantly increased at 1 h (Fig. 5B). Pyridine-2,3-dicarboxylate is the biosynthetic precursor to niacin and acts as a pro-oxidant.

#### Riboflavin metabolism

Riboflavin metabolism is the main hub for the production of FMN and FAD which are employed as cofactors in a plethora of cellular flavoprotein enzymatic reactions (45, 46). The deletion of the *ribE* gene, which encodes riboflavin synthetase, is associated with the development of nitrofurantoin resistance in Gram-negative bacteria; this comes about due to the abolition of synthesis of riboflavin/FMN, a crucial cofactor for nitroreductases NfsA and NfsB (21).

**Fig 6:**
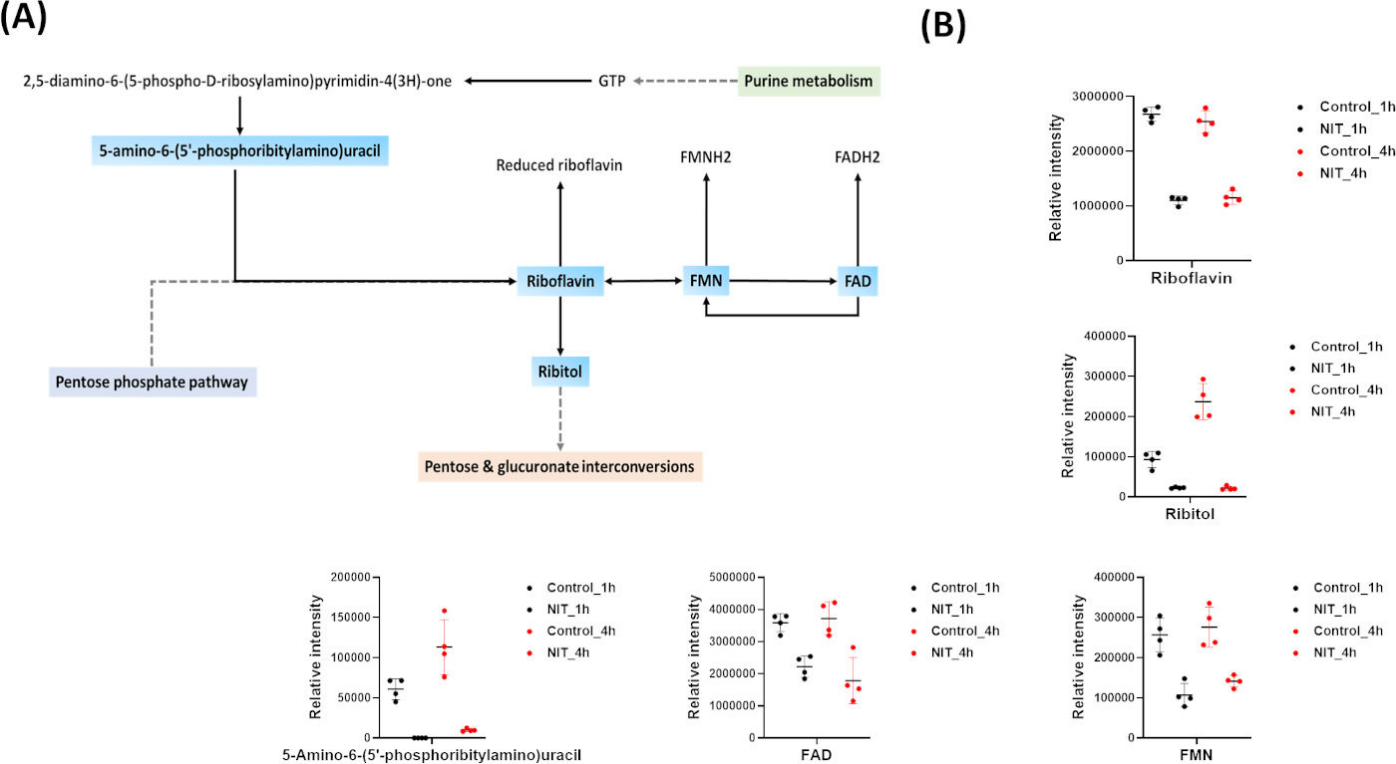
(**A**) The significantly perturbed riboflavin metabolism of *K. pneumoniae* INF348 due to nitrofurantoin treatment. (**B**) Bar charts for the significantly perturbed intermediates from riboflavin metabolism at 1 and 4 h (log_2_FC ≥ 1.0, *P* ≤ 0.05). Data are mean ± SD for four biological replicates. Blue rectangles: significantly decreased metabolites; red rectangles: significantly increased metabolites.

Nitrofurantoin treatment produced a profound depletion in the main precursors of riboflavin biosynthesis at both 1 and 4 h (Fig. 6A and B). The abundance of riboflavin (log_2_FC = −1.28), FMN (log_2_FC = −1.26), FAD (log_2_FC = −0.69), ribitol (log_2_FC = −2.0), and 5-amino-6-(5'-phosphoribitylamino)uracil (log_2_FC = −15.89) all underwent a significant decline following nitrofurantoin treatment at 1 h (Fig. 6B). Intriguingly, the inhibitory impact of nitrofurantoin on riboflavin metabolism continued at 4 h, wherein riboflavin, FMN, FAD, ribitol, and 5-amino-6-(5'-phosphoribitylamino)uracil experienced a marked depletion following nitrofurantoin therapy (log_2_FC ≤ −1.0, *P* ≤ 0.05; Fig. 6A and B). The observed inhibitory effects of nitrofurantoin on the riboflavin biosynthetic pathway would result in a cascade that begins with a marked decrease of cellular riboflavin, which would in turn attenuate the activity of NfsA and NfsB and as such culminate as a decrease in the levels of activated nitrofurantoin intermediates (21).

### Conclusions

The study is the first to integrate the genomics and metabolomics platforms to investigate the genes that govern nitrofurantoin resistance as well as the potential metabolic pathways underlying the bacterial killing effect in *K. pneumoniae*. The comparative genomics analysis showed for the first time that nitrofurantoin resistance in *K. pneumoniae* spans across two major resistance levels, first, by limiting the amount of drug accumulating inside the bacterial cell (enhancing drug efflux and potentially also by repressing influx); and second, inhibition of FMN cofactor synthesis and mutations targeting NfsB nitroreductase that abolish its ability to enzymatically activate nitrofurantoin. The metabolomics findings suggested that nitrofurantoin exerts its antibacterial killing effects by inhibiting aminoacyl-tRNA biosynthesis and purine metabolism, which ultimately terminates bacterial ribosomal RNA, DNA, and protein synthesis.

## MATERIALS AND METHODS

### Bacterial strains

*K. pneumoniae* INF348 was cultured from the urine of a female patient in her 40s at the Alfred Hospital in Melbourne, in 2014, as part of routine diagnostic procedures. Its complete genome sequence (GenBank accession, GCA_904863425) was previously reported (47) and belongs to multi-locus sequence type (ST) 34 and carries capsule (K) locus KL22 and no acquired antimicrobial resistance or hypervirulence genes. The isolate was stored in tryptone soy broth (Oxoid) with 20% glycerol (Ajax Finechem, Seven Hills, NSW, Australia) in cryovials at −80°C. It was sub-cultured onto nutrient-rich agar plates (Media Preparation Unit, University of Melbourne, Melbourne, VIC, Australia) and incubated at 37°C for 24 h prior to use.

### Antibiotics and reagents

Mueller-Hinton broth (Oxoid, England) adjusted with calcium and magnesium (20–25 mg/L Ca^2+^ and 10–12.5 mg/L Mg^2+^) was employed for susceptibility testing and all microbiological studies. Stock solutions of nitrofurantoin (Sigma Aldrich, Australia, CAS# 67–20-9) solutions were prepared in *N,N*-dimethylformamide (DMF; Sigma Aldrich, Australia, CAS# 68–12-2). Nitrofurantoin solutions were filter sterilized using a 0.22 µm filter (Sartorius, Australia).

### Determination of MICs

MICs were assessed in triplicates using the broth microdilution method as described earlier (48). A final concentration of DMF in the growth media of 0.5% (vol/vol) showed no inhibitory effect on the tested strains.

### Induction and selection of nitrofurantoin-resistant strains

Two sequential selection steps were employed to generate nitrofurantoin-resistant *K. pneumoniae* derived from isolate INF348. After preparing the overnight culture, a fresh bacterial solution was prepared by adjusting the overnight OD_600_ value to achieve an inoculum size of OD_600_ ~ 0.1. Then 100 µL was taken and distributed evenly onto CAMHB plates containing 64 mg/L nitrofurantoin (2× MIC) and incubated for 24–48 h at 37°C. The surviving bacterial colonies were collected and then verified as resistant on the CAMHB plates containing the same concentration of nitrofurantoin. The surviving mutants were then progressed to the second selection round, wherein an overnight culture was prepared from each mutant, and 100 µL was spread evenly into CAMHB plates containing a higher nitrofurantoin concentration of 256 mg/L and incubated for 24 and 48 h. The surviving colonies were then verified as highly resistant on the 256 mg/L nitrofurantoin CAMHB plates.

### Whole-genome sequencing

The genomes of the six nitrofurantoin-resistant mutants (INF348-01, INF348-02, INF348-03, INF348-04, INF348-05, and INF348-06) and nitrofurantoin-susceptible parent strain *K. pneumoniae* INF348 were sequenced on an Illumina HiSeq instrument (Illumina Australia, Melbourne, VIC, Australia) to over 30× coverage, following Nextera library preparation. INF348 was also subjected to long-read sequencing via Oxford Nanopore Technologies as previously described (49). The Illumina short and Nanopore long reads for INF348 were used to generate a hybrid assembly using Unicycler v0.4.7 using default parameters, and the resulting completed genome was annotated with Prokka v1.13.3 (50). Illumina short reads for each mutant were mapped to the completed INF348 genome using RedDog to detect single nucleotide variants (SNVs; available at https://github.com/katholt/RedDog). Briefly, RedDog employs Bowtie v2.2.9 (51) with a sensitive local algorithm and a maximum insert length of 2,000 bp, and variant sites are called with SAMtools v1.3.1 (52). RedDog also produces depth and coverage statistics for each gene in the reference genome; these statistics were interrogated to identify missing genes relative to INF348 in each mutant genome. Genes were considered missing if the coverage across the gene was <95%, or the read depth was <5×. The completed INF348 genome was screened for IS using ISSaga (53). Seven IS types were identified in INF348 (IS*1203*, IS*1 × 2*, IS*903B*, ISEc52, ISKpn21, ISKpn26, and ISSty2). Illumina readsets for all mutant genomes were screened for these seven IS using ISMapper v2.0 (54) with default parameters and the INF348 reference genome. IS insertion sites that varied from INF348, as well as detected gene deletions, were annotated in each respective mutant genome and visually inspected using Artemis (55). All genes carrying mutations (SNVs, deletions, or IS insertions) not present in the founder strain were identified using BLAST searches of the NCBI database and by consulting the literature.

### RNA extraction and reverse transcription

The six nitrofurantoin-resistant isolates (INF348-01, INF348-02, INF348-03, INF348-04, INF348-05, and INF348-06) and nitrofurantoin-susceptible parent strain *K. pneumoniae* INF348 were cultured on CAMHB plates overnight. The total RNA was extracted using RNAdvance Tissue Kit (Beckman Coulter, U.S.A), and contaminating DNA was removed using DNaseI (ThermoFisher Scientific) according to the manufacturer’s protocol. The extracted RNA was stored in −80°C for further use. The cDNA was synthesized using 4 µL of the reverse transcription buffer with random primers (Promega), 2 µL of GoScript reverse transcriptase, and 100 ng of RNA, according to the manufacturer’s protocol.

### Quantitative polymerase chain reaction

To measure the relative mRNA levels, qPCR was used to detect the fluorescent signal of the BRYT Green Dye binding to double-stranded DNA [fold change (FC) ≥2, relative to the wildtype progenitor]. The PCR mixture (20 µL) consists of 16 µL of Go Taq qPCR Master Mix, 2 µL of 0.8 µM each primer, and 2 µL of cDNA. The PCR cycle reaction was performed using a PCR Thermal cycler (ThermoFisher Scientific) under the following conditions: 95°C for 2 min for start, then 95°C for 15 seconds, 60°C for 1 min, and run 40 cycles, according to manufacturer’s protocol. The amplification efficiency of each primer pairs was tested; all the values were within 90%–100%. All primers are listed in (Table S3), and *rpoB* was used as a reference gene.

### Molecular modeling

Utilizing the ICM-Homology modeling algorithm and refinement tools (56–58) available in the ICM-Pro modeling suite (Molsoft LLC; molsoft.com), the target sequence of *K. pneumouniae* NfsB was constructed. ICM-Pro was also used for template searches for our candidate protein, allowing for automated alignment and inspection, prior to modeling the target protein. Mutational analysis focusing on protein stability was also carried using ICM-Pro.

### Bacterial culture preparation for metabolomics

*K. pneumoniae* INF348 was cultured on Mueller-Hinton agar and incubated overnight at 37°C for 20 h. Prior to each experiment, an overnight culture was prepared by inoculation of one colony in 10 mL of CAMHB in 50 mL Falcon tubes (Thermo Fisher, Australia), which were immediately incubated at 37°C in a shaking water bath (180 rpm). The following day, a fresh bacterial solution was prepared in a 500 mL conical flask containing fresh CAMHB, which was incubated at 37°C at a shaking speed of 180 rpm for 2 h to achieve a log-phase of OD600 ~0.5 (~10^8^ colony-forming units (CFU) per mL). Freshly prepared nitrofurantoin solution in milliQ water was then added to the correspondence flask to give a final concentration of 48 mg/L (1.5× MIC), and a drug-free control flask was also prepared. The flasks were returned to the rotary shaker at 37°C and a shaking speed of 180 rpm. At each time point (1 and 4 h), 15 mL of each sample was collected, quenched, and normalized to the required OD600 ~ 0.5 with fresh CAMHB. Thereafter, the samples were centrifuged at 3,220 × *g* at 4°C for 10 min. Lastly, the supernatants were discarded, and the pellets were stored at −80°C for further metabolite extraction. The experiment was conducted in four biological replicates to minimize the bias from inherent random variation.

### Cellular metabolite extraction

The bacterial pellets were rinsed with cold 0.9% NaCl followed by centrifugation at 3,220 *× g* at 4°C for 5 min to eliminate residual extracellular metabolites. Extraction solvent of chloroform:methanol:water [1:3:1, (vol/vol)] containing 1 µM of generic internal standards (CHAPS, CAPS, PIPES, and TRIS) was then added. The samples were immediately frozen in liquid nitrogen and allowed to thaw on ice, and the freeze-thaw process was repeated three times to lyse the cells and release cellular metabolites. The extracted samples were centrifuged for 10 min at 3,220 *× g* at 4°C, and the supernatant was collected and further centrifuged at 14,000 *× g* for 10 min at 4°C. The final supernatant samples (200 µL) were collected into injector vials for Liquid Chromatography–Mass Spectrometry (LC-MS) analysis. Quality control samples were prepared by combining an equal volume of each sample (59).

### LC-MS analysis of metabolites

Samples were analyzed on a Q-Exactive Orbitrap mass spectrometer (Thermo Fisher), coupled to a Dionex high-performance liquid chromatograph (U3000 RSLC HPLC, Thermo Fisher) with a ZIC-pHILIC column (5 µm, polymeric, 150 × 4.6 mm; SeQuant, Merck). The MS system operated at 35,000 resolutions in both positive and negative electro-spray ionization mode (rapid switching) and a detection range of 85–1,275 m/z. The LC solvent consisted of 20 mM ammonium carbonate (A) and acetonitrile (B) with a multistep gradient system from 80% B to 50% B over 15 min, then to 5% B at 18 min, followed by a wash with 5% B for 3 min, and re-equilibration for 8 min with 80% B at a flow rate of 0.3 mL/min. The injection sample volume was 10 µL, and the run time was 32 min. All samples were analyzed in the same run and the chromatographic peaks, signal reproducibility, and analyte stability were monitored by assessment of pooled biological quality control samples (i.e., an aliquot of 10 µL from each sample, including both footprints and fingerprints) analyzed periodically throughout the batch, internal standards, and total ion chromatograms for each sample. Mixtures of pure standards containing over 200 metabolites were analyzed within the batch to aid in the identification of metabolites.

### Metabolomics data processing and analysis

Metabolomics data analyses were performed using mzMatch72 and IDEOM, as we have previously described in greater detail (60–63). Briefly, the quantification of each metabolite was conducted using the raw peak height. Univariate and multivariate analyses utilized MetaboAnalyst 3.074. Prior to analysis, relative peak intensity data were normalized by the median, log transformed, and scaled (by auto scale function) to reduce variance between the samples. The global metabolic profiles of samples with antibiotic treatments at each time point were analyzed using multivariate statistical analysis by the partial least square’s discriminant analysis. Univariate analysis (*t* test; *P* < 0.05, fold change threshold = 1.5) was applied to identify significant metabolite changes between treated and untreated control samples at each time point. Metabolites that were detected as isomeric peaks with opposite abundance changes (increased and decreased levels) were excluded. Significant metabolites were filtered by selection of only those that showed a ≥0.59 log_2_-FC relative to the untreated control samples and an identification confidence score of 6 or more in IDEOM, i.e., removing likely LC-MS artifacts. Metabolic pathway analysis was performed for the statistically significant identified metabolites using KEGG Mapper (64).

## SUPPLEMENTAL MATERIAL

Supplemental material msystems.00972-23-s0001.docx Supplemental figures, tables, and text.

ASM does not own the copyrights to Supplemental Material that may be linked to, or accessed through, an article. The authors have granted ASM a non-exclusive, world-wide license to publish the Supplemental Material files. Please contact the corresponding author directly for reuse.
