# Supplementary material for: High-level nitrofurantoin resistance in a clinical isolate of Klebsiella pneumoniae: a comparative genomics and metabolomics analysis
Source: mSystems. 2023 Dec 11;9(1):e00972-23. doi: 10.1128/msystems.00972-23 (PMC10805014; doi:10.1128/msystems.00972-23)
Supplement: Supplemental material — Supplemental figures, tables, and text. [file msystems.00972-23-s0001.docx]

**Supplementary Data: High-level nitrofurantoin resistance in a clinical isolate of *Klebsiella pneumoniae*: A comparative genomics and metabolomics analysis**

Maytham Hussein,^1^ Zetao Sun,^2^ Jane Hawkey,^3^ Rafah Allobawi,^1^ Louise M. Judd,^4^ Vincenzo Carbone,^5^ Rajnikant Sharma,^7^ Varsha Thrombare,^1^ Mark Baker,^8^ Gauri G. Rao,^7^ Jian Li,^10^ Kathryn E. Holt,^3,9^* Tony Velkov^1,2^*

**Affiliations:** ^1^Monash Biomedicine Discovery Institute, Department of Pharmacology, Monash University, Clayton, VIC 3800, Australia; ^2^Department of Biochemistry & Pharmacology, School of Biomedical Sciences, Faculty of Medicine, Dentistry and Health Sciences, The University of Melbourne, Parkville, VIC 3010, Australia; ^3^Department of Infectious Diseases, Central Clinical School, Monash University, Melbourne, VIC 3004, Australia; ^4^ Doherty Applied Microbial Genomics (DAMG), Peter Doherty Institute for Infection and Immunity, The University of Melbourne, Melbourne, VIC 3010, Australia; ^5^AgResearch Limited, Grasslands Research Centre, Tennent Drive, Private Bag 11008, Palmerston North 4442, New Zealand; ^7^Division of Pharmacotherapy and Experimental Therapeutics, Eshelman School of Pharmacy, University of North Carolina, Chapel Hill, NC 27599, USA; ^8^Discipline of Biological Sciences, Priority Research Centre in Reproductive Biology, Faculty of Science and IT, University of Newcastle, University Drive, Callaghan NSW, 2308, Australia; ^9^London School of Hygiene & Tropical Medicine, London, United Kingdom; ^10^Monash Biomedicine Discovery Institute, Department of Microbiology, Monash University, Clayton, VIC 3800, Australia.

*Corresponding authors: [tony.velkov@monash.edu](mailto:tony.velkov@monash.edu.au) OR Kat.Holt@lshtm.ac.uk

**Keywords.** Nitrofurantoin, *Klebsiella pneumoniae*, antimicrobial resistance, genomics metabolomics.

**Short Title:** Omics study of high-level nitrofurantoin resistance in *Klebsiella pneumoniae*

**Supplementary Figures**

**Supplementary Figure S1.** Structure-based sequence alignment of the nfsB model based on 1YKI structure produced by Molsoft. Differential amino acids of the model are highlighted in grey while the α-helical regions are indicated with tubes, and β-sheets regions with arrows. Residues that hydrogen bond to FMN are indicated with yellow boxes.


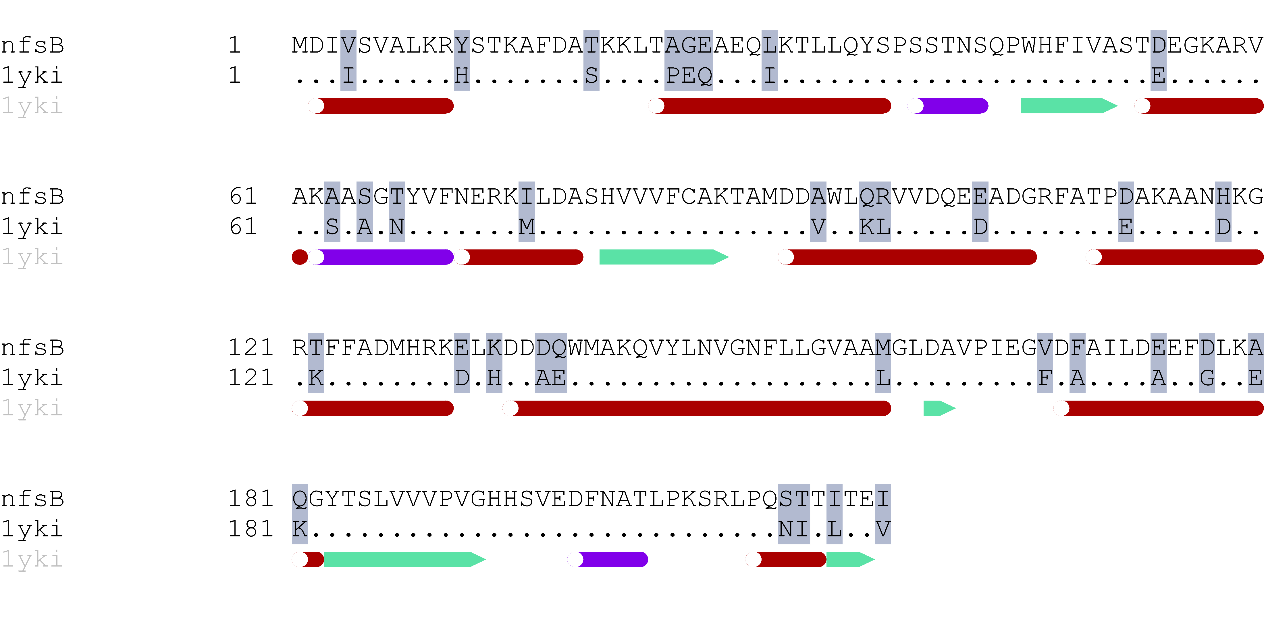


**Supplementary Figure S2.** Multivariate and univariate analyses of global metabolome changes in *K. pneumoniae* INF348 in response to nitrofurantoin treatment (48 mg/L). PLS-DA score plots for metabolite levels from samples treated with nitrofurantoin (NFT) at (**A**) 1 and (**B**) 4 h. Each data set represents a total of 16 samples of 4 biological replicates for each experimental condition. **△**= untreated control; **+** = nitrofurantoin (NFT).


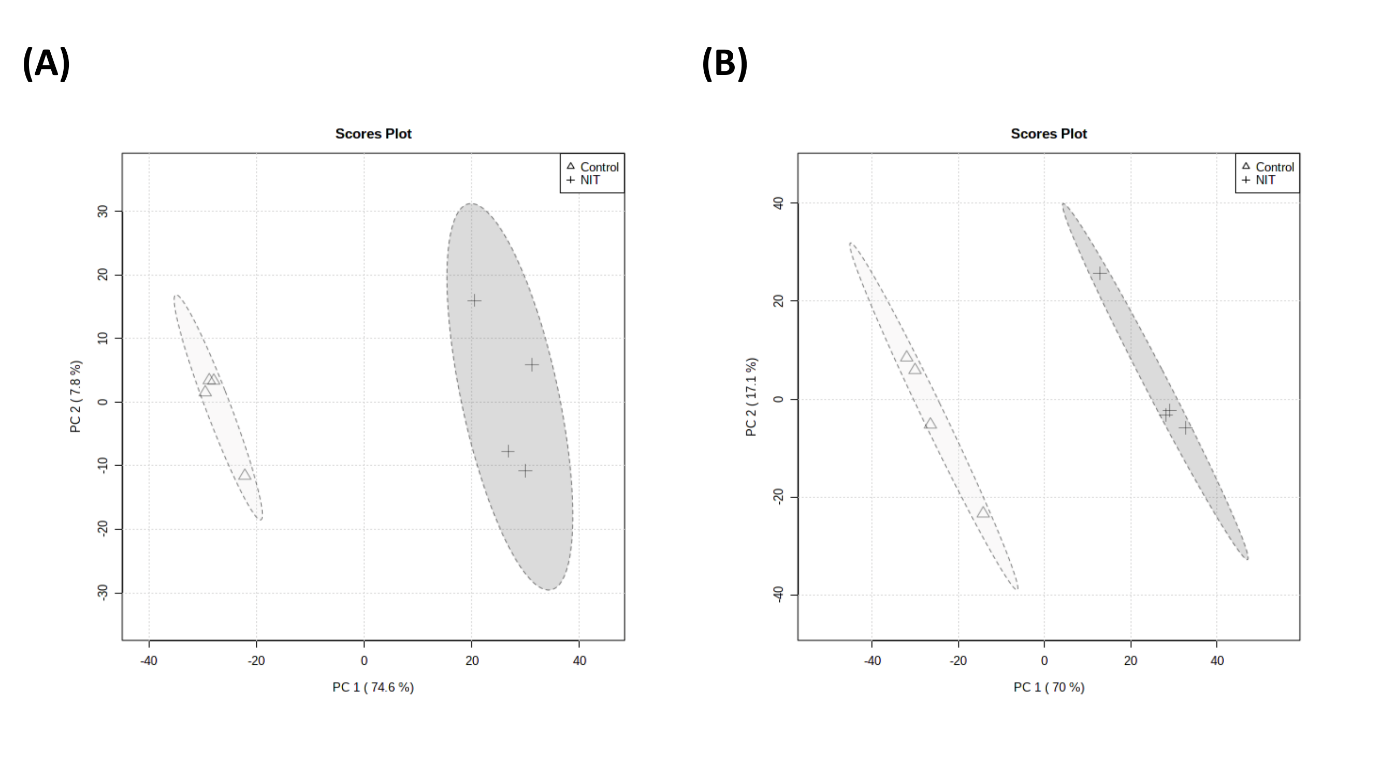


**Supplementary Figure S3.** Heatmap profiles of all identified metabolites clustered by metabolite class after treatment of *K. pneumoniae* INF348 with nitrofurantoin (NFT) (48 mg/L) at (A)1 and (B) 4 h​. Each data set represents a total of 16 samples of 4 biological replicates for each experimental condition.


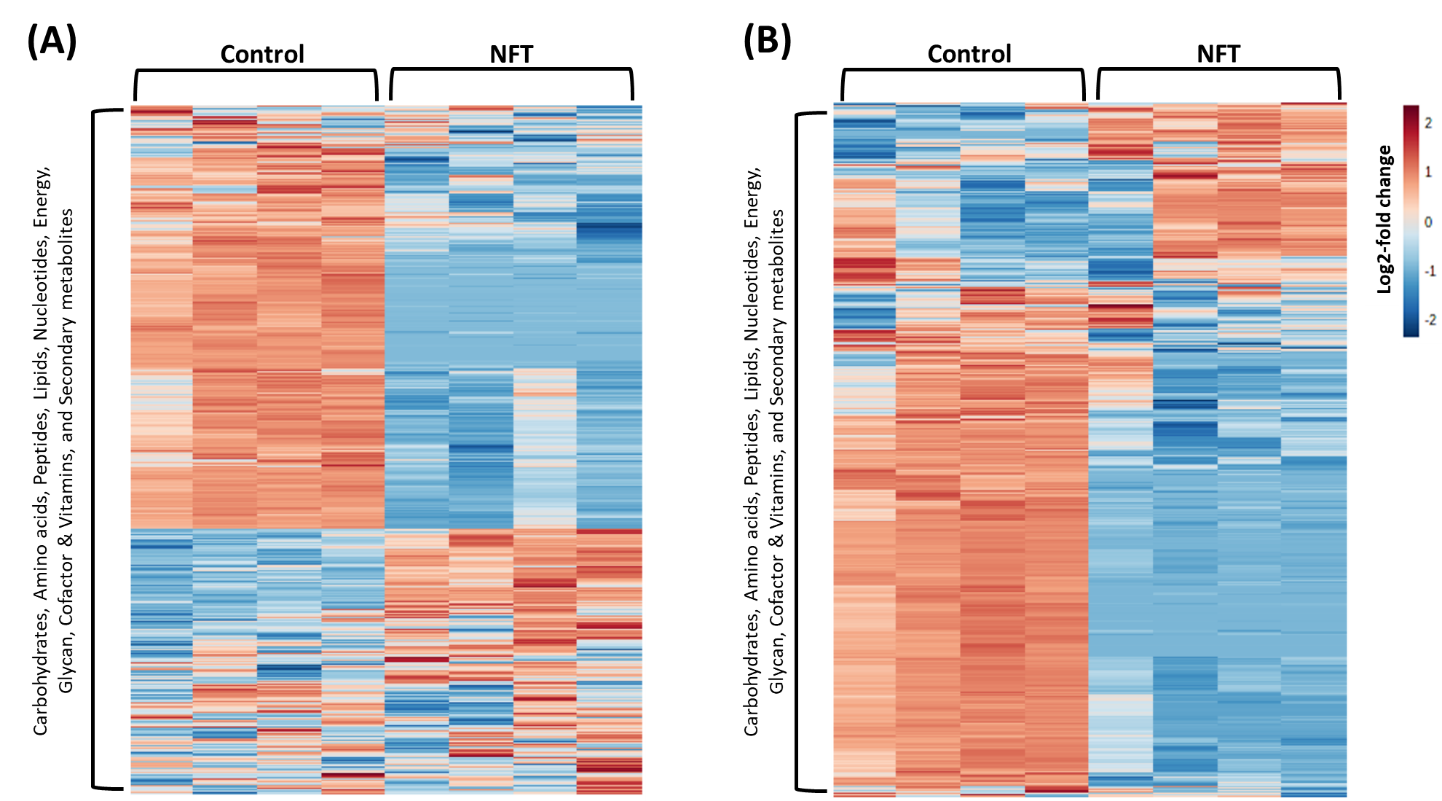


**Supplementary Figure S4.** (**A**) Total number of significant metabolites and (**B**)​ a summary of their classification according to different metabolite classes of *K. pneumoniae* INF348 treated with nitrofurantoin (NFT) (48 mg/L) at 1 and 4 h. ​(log_2_FC ≥ 0.59, *p*≤ 0.05). **(C)** Venn diagram for the significantly perturbed metabolites of *K. pneumoniae* INF348 after treatment with NFT (48 mg/L) at 1 and 4 h. ​(log_2_FC ≥ 0.59, *p*≤ 0.05). Each data set represents a total of 16 samples of 4 biological replicates for each experimental condition.


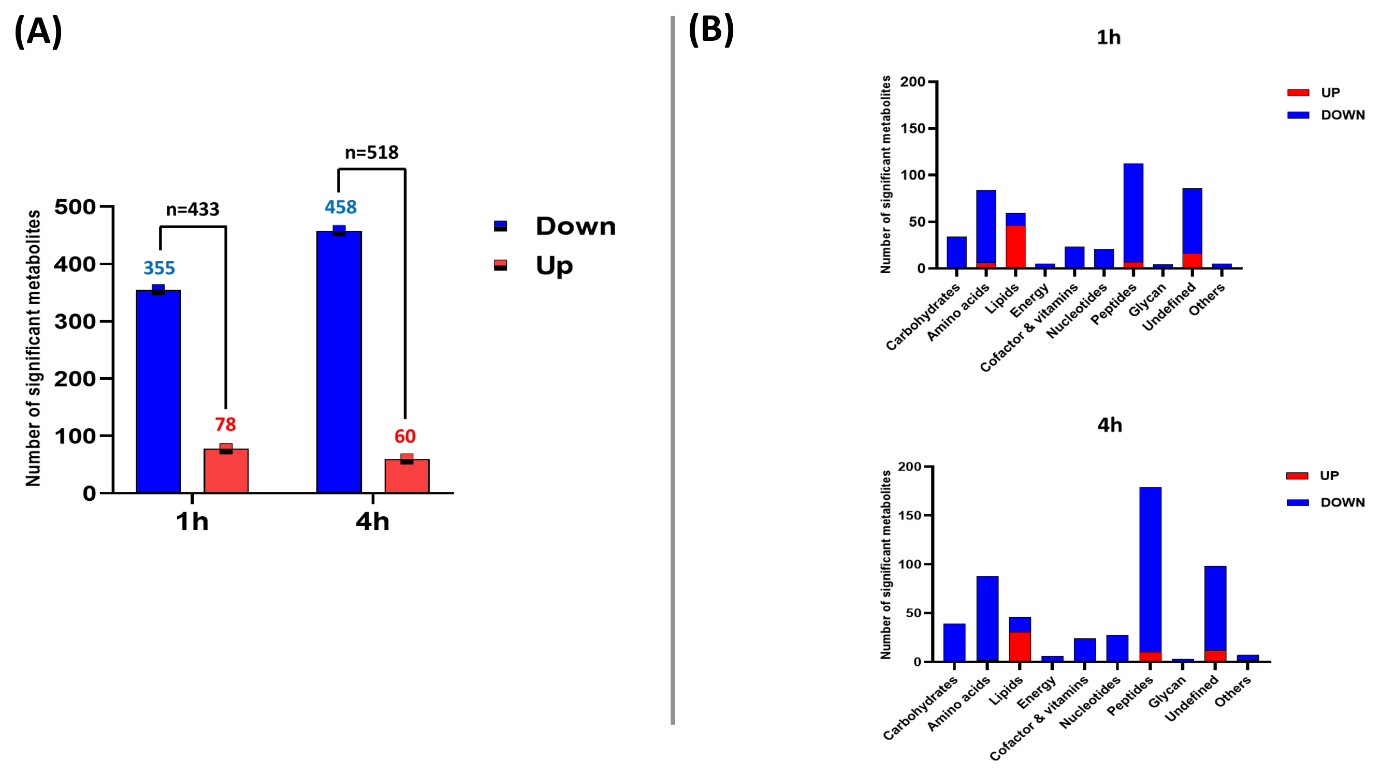


**(C)**

**
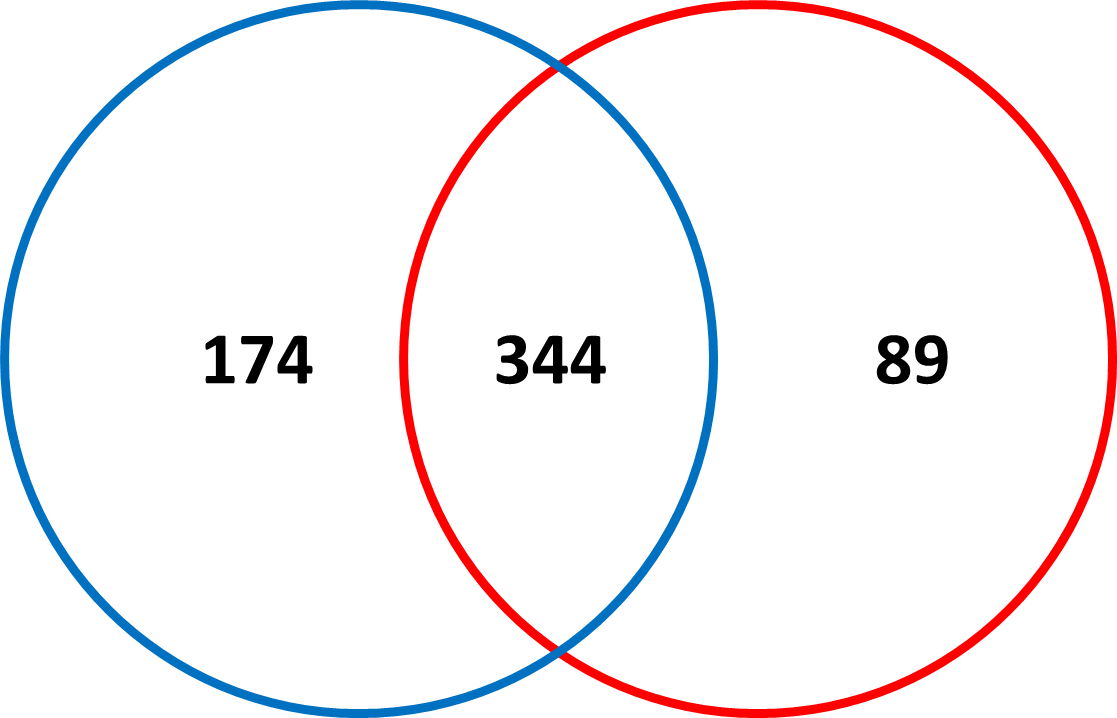
**

**Supplementary Figure S5.** Pathway view shows the significantly impacted pathways of *K. pneumoniae* INF348 after treatment with nitrofurantoin (NFT) (48 mg/L) at 1 and 4 h. ​(log_2_FC ≥ 0.59, *p*≤ 0.05). Each data set represents a total of 16 samples of 4 biological replicates for each experimental condition.


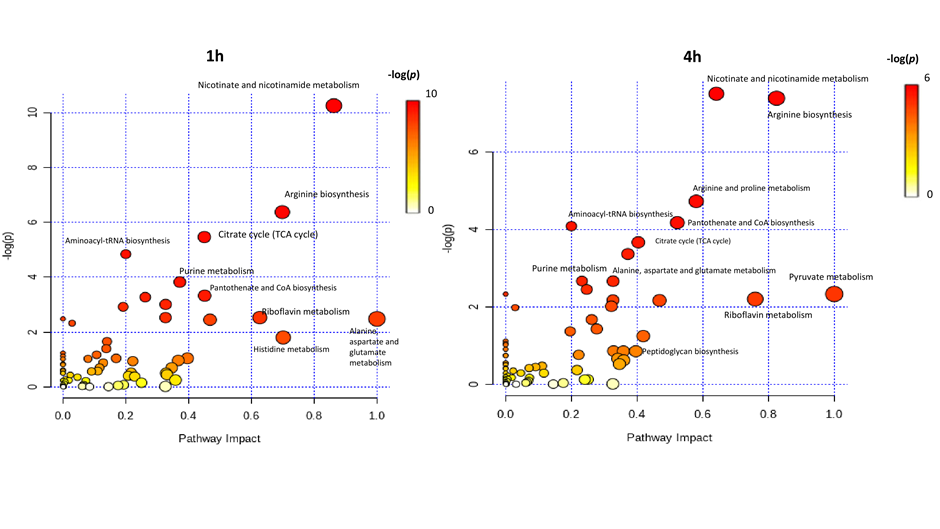


**Supplementary Figure S6.** **(A)** Graph illustrates the significantly perturbed metabolites of glycolysis and tricarboxylic acid (TCA) cycle of *K. pneumoniae* INF348 following NFT treatment at 4 h; **(B)** Bar charts for the significantly perturbed intermediates from glycolysis and TCA pathways at 1 h. (log_2_FC ≥ -1.0, *p* ≤ 0.05). Each data set represents a total of 16 samples of 4 biological replicates for each experimental condition. The grid line in the chart represents the log_2_FC cut-off of ≥ 1 or ≤ -1.

**
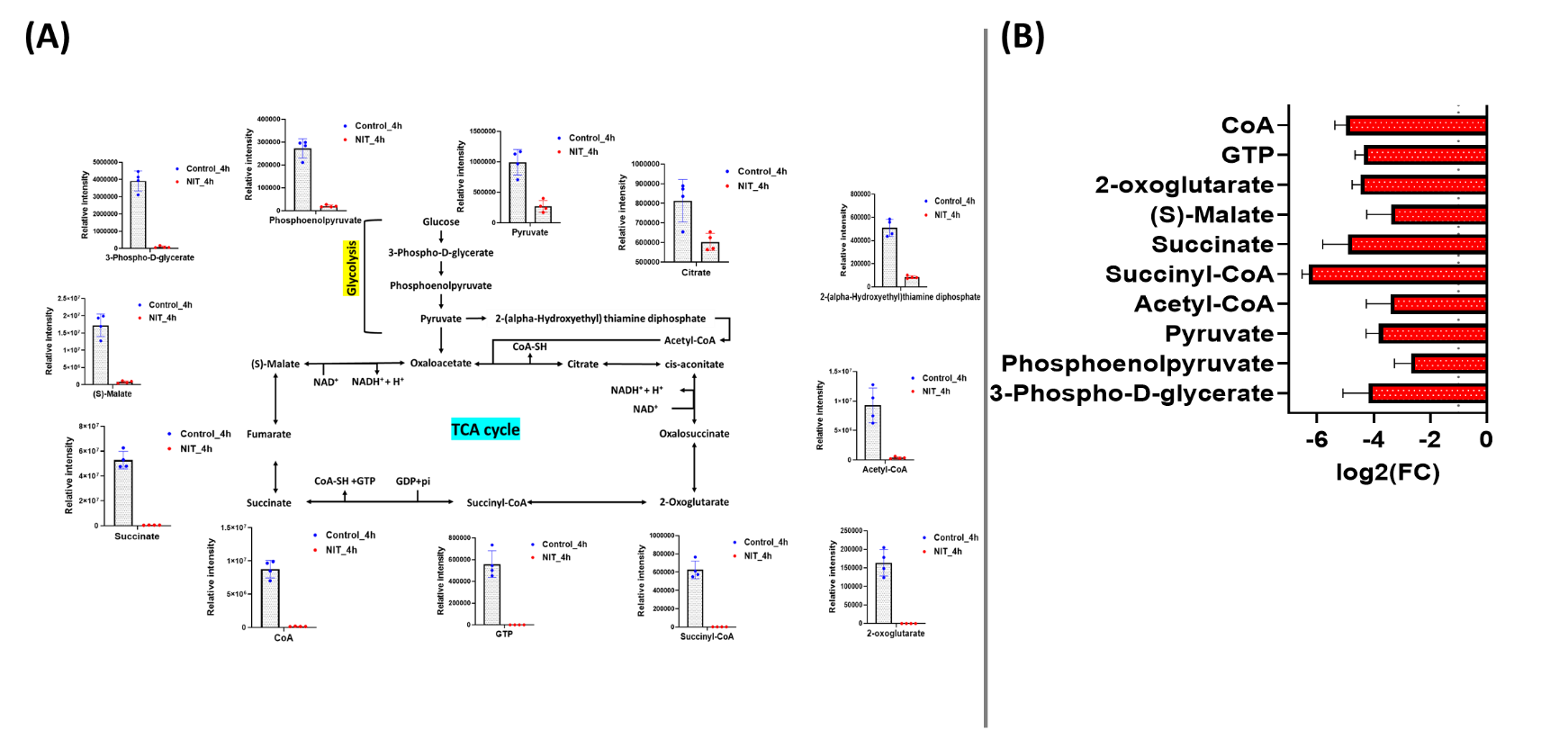
**

**Supplementary Figure S7.** Bar charts for the significantly perturbed intermediates from pantothenate and CoA biosynthesis at 1 and 4 h. (log_2_FC ≥ -1.0, *p* ≤ 0.05). Each data set represents a total of 16 samples of 4 biological replicates for each experimental condition. The grid line in the chart represents the log_2_FC cut-off of ≥ 1 or ≤ -1.

**Supplementary Tables**

**Supplementary Table S1.** Data precision of individual samples represented as the median relative standard deviation (RSD) for all metabolites of *K. pneumoniae* INF348, based on all biological replicates (*n*=4) for each group. Each data set represents a total of 16 samples of 4 biological replicates for each experimental condition.

|  | Median RSD % | |
| --- | --- | --- |
|  | **1 h** | **4 h** |
| Control | 19 | 26 |
| NIT | 18 | 26 |

**Supplementary Table S2.** Significantly impacted intermediates of *K. pneumoniae* INF348 treated with nitrofurantoin at 1 and 4 h. Significant metabolites were identified through Univariate Analysis [*t*-tests, FDR adjusted *p*-value (False Discovery Rate adjusted *p*-value) ≤ 0.05; log_2_-fold change (FC) ≥ 0.59, corresponding approximately to a 1.5-fold change in linear scale]. * Confidence levels were automatically generated in IDEOM, as described previously.^1^

| **1 hour** | | | | | |
| --- | --- | --- | --- | --- | --- |
| **Putative Metabolite** | ***p.* value** | **Map** | **Kegg ID** | ***Confidence** | **log2(FC)** |
| L-Glutamate | 5.21E-06 | Amino acid metabolism | C00025 | 8 | -2.57 |
| L-Alanine | 1.64E-06 | Amino acid metabolism | C00041 | 6 | -2.04 |
| L-Lysine | 3.91E-11 | Amino acid metabolism | C00047 | 8 | -2.04 |
| Glutathione | 1.08E-10 | Amino acid metabolism | C00051 | 8 | -4.64 |
| L-Arginine | 6.62E-06 | Amino acid metabolism | C00062 | 8 | -1.46 |
| L-Glutamine | 5.59E-09 | Amino acid metabolism | C00064 | 6 | -3.74 |
| L-Serine | 4.21E-05 | Amino acid metabolism | C00065 | 8 | -1.74 |
| L-Methionine | 3.32E-07 | Amino acid metabolism | C00073 | 8 | -1.68 |
| L-Ornithine | 5.3E-08 | Amino acid metabolism | C00077 | 8 | -2.40 |
| L-Tryptophan | 9.87E-07 | Amino acid metabolism | C00078 | 6 | -1.42 |
| L-Phenylalanine | 2.53E-06 | Amino acid metabolism | C00079 | 8 | -1.14 |
| L-Tyrosine | 2.42E-07 | Amino acid metabolism | C00082 | 8 | -1.74 |
| Choline | 0.002195 | Amino acid metabolism | C00114 | 8 | -1.11 |
| L-Leucine | 5.32E-08 | Amino acid metabolism | C00123 | 8 | -2.00 |
| L-Histidine | 0.000067 | Amino acid metabolism | C00135 | 6 | -1.60 |
| 3-Methyl-2-oxobutanoic acid | 5.74E-05 | Amino acid metabolism | C00141 | 6 | -1.41 |
| L-Proline | 9.39E-07 | Amino acid metabolism | C00148 | 8 | -1.74 |
| Phenylpyruvate | 1.48E-07 | Amino acid metabolism | C00166 | 8 | -2.27 |
| 5'-Methylthioadenosine | 0.004495 | Amino acid metabolism | C00170 | 8 | 1.31 |
| L-Valine | 4.1E-13 | Amino acid metabolism | C00183 | 8 | -4.22 |
| L-Threonine | 0.000305 | Amino acid metabolism | C00188 | 6 | -1.84 |
| 4-Methyl-2-oxopentanoate | 9.72E-06 | Amino acid metabolism | C00233 | 6 | -1.45 |
| L-Citrulline | 6.96E-16 | Amino acid metabolism | C00327 | 6 | -8.79 |
| L-Kynurenine | 0.028586 | Amino acid metabolism | C00328 | 6 | -1.44 |
| Indolepyruvate | 2.67E-08 | Amino acid metabolism | C00331 | 6 | -2.57 |
| N-Acetylornithine | 0.000294 | Amino acid metabolism | C00437 | 6 | -3.94 |
| N-Formimino-L-glutamate | 6.27E-08 | Amino acid metabolism | C00439 | 8 | -3.48 |
| N6-(L-1,3-Dicarboxypropyl)-L-lysine | 0.016233 | Amino acid metabolism | C00449 | 5 | 1.32 |
| N-Acetyl-L-glutamate | 6.43E-14 | Amino acid metabolism | C00624 | 8 | -3.14 |
| 5-Hydroxy-L-tryptophan | 9.74E-08 | Amino acid metabolism | C00643 | 5 | -1.28 |
| LL-2,6-Diaminoheptanedioate | 0.00165 | Amino acid metabolism | C00666 | 6 | -1.98 |
| gamma-L-Glutamyl-L-cysteine | 0.000279 | Amino acid metabolism | C00669 | 6 | -4.43 |
| Urocanate | 4.27E-08 | Amino acid metabolism | C00785 | 8 | -2.85 |
| Creatinine | 0.003679 | Amino acid metabolism | C00791 | 7 | -1.13 |
| Pantothenate | 2.05E-13 | Amino acid metabolism | C00864 | 6 | -3.98 |
| L-2-Aminoadipate | 2.06E-09 | Amino acid metabolism | C00956 | 6 | -1.93 |
| 5-Aminopentanamide | 2.11E-07 | Amino acid metabolism | C00990 | 5 | -3.47 |
| D-Alanyl-D-alanine | 1.19E-07 | Amino acid metabolism | C00993 | 6 | -2.83 |
| 4-Guanidinobutanoate | 7.4E-08 | Amino acid metabolism | C01035 | 8 | -1.88 |
| UDP-N-acetylmuramate | 1.06E-14 | Amino acid metabolism | C01050 | 6 | -5.67 |
| O-Succinyl-L-homoserine | 7.85E-11 | Amino acid metabolism | C01118 | 6 | -4.10 |
| N(pi)-Methyl-L-histidine | 0.02258 | Amino acid metabolism | C01152 | 5 | -1.51 |
| 3-(4-Hydroxyphenyl)pyruvate | 9.78E-09 | Amino acid metabolism | C01179 | 8 | -3.13 |
| 3-2-Hydroxyphenylpropanoate | 7.5E-11 | Amino acid metabolism | C01198 | 5 | -7.23 |
| 3-(2-Hydroxyphenyl)propanoate | 1.26E-07 | Amino acid metabolism | C01198 | 7 | -5.41 |
| 1-Aminocyclopropane-1-carboxylate | 1.32E-06 | Amino acid metabolism | C01234 | 8 | -2.86 |
| 4-(2-Aminophenyl)-2,4-dioxobutanoate | 1.04E-05 | Amino acid metabolism | C01252 | 6 | -1.24 |
| 4-Oxoproline | 0.000144 | Amino acid metabolism | C01877 | 7 | -1.01 |
| L-Histidinal | 1.41E-06 | Amino acid metabolism | C01929 | 6 | -1.76 |
| Indolelactate | 2.07E-10 | Amino acid metabolism | C02043 | 7 | -6.94 |
| O-Acetylcarnitine | 0.014201 | Amino acid metabolism | C02571 | 5 | -3.46 |
| N-Acetylputrescine | 1.32E-12 | Amino acid metabolism | C02714 | 6 | -3.01 |
| N6-Acetyl-L-lysine | 1.12E-09 | Amino acid metabolism | C02727 | 5 | -1.08 |
| N6-Methyl-L-lysine | 1.83E-08 | Amino acid metabolism | C02728 | 7 | -1.75 |
| L-Lysine 1,6-lactam | 4.4E-09 | Amino acid metabolism | C02837 | 7 | -2.48 |
| 4-Acetamidobutanoate | 2.65E-09 | Amino acid metabolism | C02946 | 6 | -1.20 |
| 5-Acetamidopentanoate | 1.01E-05 | Amino acid metabolism | C03087 | 5 | -1.29 |
| N-Formyl-L-methionine | 6.83E-19 | Amino acid metabolism | C03145 | 5 | -7.34 |
| Imidazol-5-yl-pyruvate | 1.73E-08 | Amino acid metabolism | C03277 | 5 | -2.59 |
| N2-Succinyl-L-arginine | 1.37E-10 | Amino acid metabolism | C03296 | 8 | -11.86 |
| L-Tyrosine methyl ester | 0.010778 | Amino acid metabolism | C03404 | 5 | -2.32 |
| N-(L-Arginino)succinate | 2.01E-15 | Amino acid metabolism | C03406 | 6 | -5.76 |
| N2-Succinyl-L-ornithine | 2.63E-07 | Amino acid metabolism | C03415 | 6 | -6.42 |
| N-Acetyl-L-phenylalanine | 0.000344 | Amino acid metabolism | C03519 | 5 | -2.89 |
| 4-Imidazolone-5-propanoate | 1.15E-05 | Amino acid metabolism | C03680 | 6 | -6.47 |
| Pyridine-2,3-dicarboxylate | 0.000048 | Amino acid metabolism | C03722 | 8 | 1.38 |
| 5-Guanidino-2-oxopentanoate | 2.03E-08 | Amino acid metabolism | C03771 | 5 | -1.44 |
| N2-(D-1-Carboxyethyl)-L-lysine | 0.000019 | Amino acid metabolism | C04020 | 7 | -5.37 |
| N2-(D-1-Carboxyethyl)-L-arginine | 1.87E-14 | Amino acid metabolism | C04137 | 5 | -5.34 |
| alpha-N-Phenylacetyl-L-glutamine | 0.000039 | Amino acid metabolism | C04148 | 5 | -1.01 |
| UDP-N-acetylmuramoyl-L-alanyl-D-glutamyl-6-carboxy-L-lysyl-D-alanyl- D-alanine | 1.07E-06 | Amino acid metabolism | C04882 | 8 | -7.79 |
| (1-Ribosylimidazole)-4-acetate | 0.003098 | Amino acid metabolism | C05131 | 5 | -2.59 |
| Hydantoin-5-propionate | 1.56E-15 | Amino acid metabolism | C05565 | 5 | -6.06 |
| Hercynine | 2.7E-08 | Amino acid metabolism | C05575 | 5 | -1.20 |
| 3,4-Dihydroxymandelate | 0.000121 | Amino acid metabolism | C05580 | 6 | -3.67 |
| 3-Methoxy-4-hydroxyphenylglycolaldehyde | 1.25E-11 | Amino acid metabolism | C05583 | 6 | -7.50 |
| Phenylpropanoate | 0.027704 | Amino acid metabolism | C05629 | 5 | 1.29 |
| 5-Hydroxyindoleacetate | 0.002512 | Amino acid metabolism | C05635 | 6 | 1.05 |
| N4-Acetylaminobutanal | 0.011936 | Amino acid metabolism | C05936 | 6 | -1.30 |
| (S)-2-Aceto-2-hydroxybutanoate | 0.018866 | Amino acid metabolism | C06006 | 6 | 1.75 |
| Hydroxymethylphosphonate | 2.89E-05 | Amino acid metabolism | C06455 | 7 | -7.10 |
| 3-(Methylthio)propionic acid | 2.88E-05 | Amino acid metabolism | C08276 | 6 | -5.36 |
| 1,2-Dihydroxy-5-(methylthio)pent-1-en-3-one | 2.86E-05 | Amino acid metabolism | C15606 | 6 | -8.07 |
| gamma-Glutamyl-gamma-aminobutyraldehyde | 1.21E-07 | Amino acid metabolism | C15700 | 5 | -1.85 |
| 6-endo-Hydroxycineole | 0.031711 | Biosynthesis of Secondary metabolite | C03092 | 5 | 1.58 |
| 7-O-Acetylsalutaridinol | 0.001864 | Biosynthesis of Secondary metabolite | C05322 | 5 | -2.69 |
| Deoxyguanidinoproclavaminic acid | 1.49E-08 | Biosynthesis of Secondary metabolite | C06656 | 6 | -3.05 |
| Proclavaminic acid | 7.35E-08 | Biosynthesis of Secondary metabolite | C06658 | 6 | -2.27 |
| 5-Acetylamino-6-formylamino-3-methyluracil | 1.46E-17 | Biosynthesis of Secondary metabolite | C16365 | 7 | 8.85 |
| CoA.1 | 1.38E-12 | Carbohydrate metabolism | C00010 | 8 | -7.69 |
| C+191:224oA | 4.76E-12 | Carbohydrate metabolism | C00010 | 8 | -4.98 |
| Pyruvate | 2.36E-11 | Carbohydrate metabolism | C00022 | 6 | -3.85 |
| Acetyl-CoA | 0.001935 | Carbohydrate metabolism | C00024 | 8 | -7.82 |
| 2-Oxoglutarate | 5.17E-05 | Carbohydrate metabolism | C00026 | 8 | -5.88 |
| UDP-glucose | 5.12E-15 | carbohydrate metabolism | C00029 | 6 | -8.16 |
| Succinate | 3.06E-11 | Carbohydrate metabolism | C00042 | 8 | -4.87 |
| Phosphoenolpyruvate | 9.29E-07 | Carbohydrate metabolism | C00074 | 6 | -4.51 |
| Succinyl-CoA | 6.89E-14 | Carbohydrate metabolism | C00091 | 6 | -7.71 |
| D-Fructose | 4.14E-10 | Carbohydrate metabolism | C00095 | 8 | -3.96 |
| D-Ribose | 0.000224 | Carbohydrate metabolism | C00121 | 8 | -1.44 |
| (S)-Malate | 5.7E-08 | Carbohydrate metabolism | C00149 | 8 | -3.33 |
| Citrate | 0.002188 | Carbohydrate metabolism | C00158 | 8 | -1.77 |
| UDP-glucuronate | 5.16E-08 | Carbohydrate metabolism | C00167 | 8 | -8.41 |
| S-Lactate | 5.9E-07 | Carbohydrate metabolism | C00186 | 6 | -2.63 |
| 3-Phospho-D-glycerate | 2.65E-07 | Carbohydrate metabolism | C00197 | 8 | -4.13 |
| D-Glucono-1,5-lactone | 5.47E-08 | Carbohydrate metabolism | C00198 | 6 | -2.33 |
| D-Gluconic acid | 4.56E-05 | Carbohydrate metabolism | C00257 | 8 | -3.21 |
| D-Glycerate | 6.31E-10 | Carbohydrate metabolism | C00258 | 6 | -5.12 |
| N-Acetylneuraminate | 0.001056 | Carbohydrate metabolism | C00270 | 6 | -1.62 |
| D-Erythrose 4-phosphate | 1.13E-09 | Carbohydrate metabolism | C00279 | 6 | -2.74 |
| D-Glucosamine | 4.77E-16 | Carbohydrate metabolism | C00329 | 7 | -6.42 |
| Mannitol | 2.71E-07 | Carbohydrate metabolism | C00392 | 6 | -4.69 |
| cis-Aconitate | 1.09E-06 | Carbohydrate metabolism | C00417 | 6 | -1.84 |
| D-Xylonate | 0.000103 | Carbohydrate metabolism | C00502 | 7 | -1.71 |
| 2-Deoxy-D-ribose 1-phosphate | 6.62E-07 | Carbohydrate metabolism | C00672 | 8 | -4.06 |
| 2-Acetolactate | 3.4E-10 | Carbohydrate metabolism | C00900 | 6 | -3.82 |
| 6-Phospho-2-dehydro-D-gluconate | 1.75E-05 | Carbohydrate metabolism | C01218 | 6 | -4.19 |
| D-Erythrose | 1.58E-05 | Carbohydrate metabolism | C01796 | 5 | -1.19 |
| 3-Ethylmalate | 0.00011 | Carbohydrate metabolism | C01989 | 5 | -5.77 |
| (R)-S-Lactoylglutathione | 7.27E-07 | Carbohydrate metabolism | C03451 | 6 | -8.03 |
| 1-O-Methyl-myo-inositol | 2.94E-11 | Carbohydrate metabolism | C03659 | 7 | -3.89 |
| D-Sedoheptulose 7-phosphate | 2.6E-15 | Carbohydrate metabolism | C05382 | 6 | -7.38 |
| dTDP-4-acetamido-4_6-dideoxy-D-glucose | 2.78E-07 | Carbohydrate metabolism | C06018 | 5 | -3.63 |
| ATP | 6.21E-13 | Energy Metabolism | C00002 | 6 | -6.35 |
| NAD | 1.52E-11 | Energy Metabolism | C00003 | 8 | -5.17 |
| NADP | 1.41E-06 | Energy Metabolism | C00006 | 6 | -6.00 |
| Orthophosphate | 0.000801 | Energy Metabolism | C00009 | 7 | -3.32 |
| Sedoheptulose | 1.86E-08 | Energy Metabolism | C02076 | 5 | -5.07 |
| 9-methoxy-pentadecanoic acid | 0.019952 | Fatty Acyls[FA] | NA | 5 | 1.73 |
| (3R)-oct-1-en-3-ol | 8.34E-06 | Fatty Acyls[FA] | NA | 5 | 1.79 |
| 9-hydroxy-12Z-octadecenoic acid | 0.009723 | Fatty Acyls[FA] | NA | 5 | 2.41 |
| 9-hydroxy-10E,12Z-octadecadienoic acid | 0.02646 | Fatty Acyls[FA] | NA | 5 | 2.46 |
| 9-methoxy-heptadecanoic acid | 0.007042 | Fatty Acyls[FA] | NA | 5 | 2.84 |
| 3-Deoxy-D-manno-octulosonate | 7.33E-10 | Glycan Biosynthesis and Metabolism | C01187 | 6 | -7.19 |
| UDP-3-O-(3-hydroxytetradecanoyl)-N-acetylglucosamine | 0.000404 | Glycan Biosynthesis and Metabolism | C04738 | 6 | -2.17 |
| 2,3-Bis(3-hydroxytetradecanoyl)-beta-D-glucosaminyl 1-phosphate | 2.96E-06 | Glycan Biosynthesis and Metabolism | C04824 | 6 | -3.55 |
| 2,3-Bis3-hydroxytetradecanoyl-beta-D-glucosaminyl 1-phosphate | 9.96E-08 | Glycan Biosynthesis and Metabolism | C04824 | 8 | -2.44 |
| 1-tetradecanoyl-2-(9Z-octadecenoyl)-sn-glycero-3-phosphocholine | 0.001937 | Glycerophospholipids[GP] | NA | 5 | 1.21 |
| 1+239:272-octadecanoyl-2-(9Z,12Z-octadecadienoyl)-sn-glycero-3-phosphoethanolamine | 0.003263 | Glycerophospholipids[GP] | NA | 5 | 2.52 |
| 1-pentadecanoyl-2-(9Z-hexadecenoyl)-sn-glycero-3-phosphocholine | 0.005033 | Glycerophospholipids[GP] | NA | 5 | 4.98 |
| Hexadecanoic acid | 0.009167 | Lipid Metabolism | C00249 | 8 | 3.08 |
| LPA(16:0) | 0.000466 | Lipid Metabolism | C00416 | 7 | -1.49 |
| LPA(18:1) | 0.000653 | Lipid Metabolism | C00416 | 5 | -1.24 |
| Choline phosphate | 3.44E-07 | Lipid Metabolism | C00588 | 6 | -1.09 |
| Dodecanoic acid | 0.004225 | Lipid Metabolism | C02679 | 5 | 2.10 |
| Tetradecanoic acid | 0.003902 | Lipid Metabolism | C06424 | 5 | 2.88 |
| (9Z)-Hexadecenoic acid | 0.001601 | Lipid Metabolism | C08362 | 5 | 3.84 |
| 2-C-Methyl-D-erythritol 4-phosphate | 4.85E-07 | Lipid Metabolism | C11434 | 6 | -4.48 |
| FA methyl(5:1) | 0.009568 | Lipids:Fatty Acyls | NA | 7 | 1.26 |
| FA amino(14:0) | 0.01647 | Lipids:Fatty Acyls | NA | 7 | 1.51 |
| decanamide | 0.022409 | Lipids:Fatty Acyls | NA | 5 | 1.67 |
| FA (11:0) | 0.014045 | Lipids:Fatty Acyls | NA | 5 | 1.79 |
| FA oxo(16:0) | 0.008561 | Lipids:Fatty Acyls | NA | 5 | 1.83 |
| FA (17:0) | 0.013295 | Lipids:Fatty Acyls | NA | 5 | 3.07 |
| FA (20:4) | 0.006326 | Lipids:Fatty Acyls | C00219 | 6 | 4.62 |
| FA (18:1) | 0.009598 | Lipids:Fatty Acyls | C00712 | 6 | 3.67 |
| 5-Hydroxypentanoate | 8.7E-14 | Lipids:Fatty Acyls | C02804 | 5 | -4.83 |
| FA (7:0) | 0.010624 | Lipids:Fatty Acyls | C05799 | 5 | 1.26 |
| FA (18:3) | 0.004732 | Lipids:Fatty Acyls | C06426 | 6 | 4.10 |
| 12-Hydroxydodecanoic acid | 3.56E-05 | Lipids:Fatty Acyls | C08317 | 5 | 1.79 |
| FA hydroxy(4:0) | 0.000531 | Lipids:Fatty Acyls | C11842 | 8 | 2.09 |
| omega-Cyclohexylundecanoic acid | 0.007712 | Lipids:Fatty Acyls | C12100 | 5 | 2.97 |
| FA (20:0) | 0.008833 | Lipids:Fatty Acyls | C16526 | 5 | 7.95 |
| Pentadecanoic acid | 0.004404 | Lipids:Fatty Acyls | C16537 | 5 | 3.10 |
| GL methyl(16:0/8:0) | 9.45E-05 | Lipids:Glycerolipids | NA | 5 | -5.68 |
| LysoPE(16:1) | 1.07E-05 | Lipids:Glycerophospholipid | NA | 7 | 1.39 |
| PS (18:1) | 0.008953 | Lipids:Glycerophospholipids | NA | 7 | -7.27 |
| 1,2-dihexadecanoyl-sn-glycero-3-cytidine-5'-diphosphate | 2.09E-12 | Lipids:Glycerophospholipids | NA | 5 | -5.73 |
| PG(17:1) | 0.000282 | Lipids:Glycerophospholipids | NA | 5 | -4.12 |
| PG (14:0) | 0.032249 | Lipids:Glycerophospholipids | NA | 5 | -1.83 |
| PG (18:1) | 0.00155 | Lipids:Glycerophospholipids | NA | 5 | -1.50 |
| PG(16:1) | 0.00016 | Lipids:Glycerophospholipids | NA | 7 | -1.16 |
| PG(15:0) | 0.024754 | Lipids:Glycerophospholipids | NA | 5 | 1.24 |
| LysoPE(14:1) | 0.00013 | Lipids:Glycerophospholipids | NA | 5 | 1.92 |
| PS(16:0/18:0) | 0.009251 | Lipids:Glycerophospholipids | NA | 5 | 1.94 |
| PG(13:0) | 0.007026 | Lipids:Glycerophospholipids | NA | 5 | 2.18 |
| PG (14:0/14:0) | 0.00045 | Lipids:Glycerophospholipids | NA | 5 | 2.19 |
| PG(12:0) | 0.005288 | Lipids:Glycerophospholipids | NA | 5 | 2.25 |
| 1,2-dipentadecanoyl-sn-glycero-3-phospho-(1'-sn-glycerol) | 0.003764 | Lipids:Glycerophospholipids | NA | 5 | 2.73 |
| PA(19:1) | 0.009276 | Lipids:Glycerophospholipids | NA | 5 | 2.81 |
| PG(16:0) | 0.028759 | Lipids:Glycerophospholipids | NA | 5 | 2.95 |
| PC (10:0/13:0) | 0.007268 | Lipids:Glycerophospholipids | NA | 5 | 3.93 |
| PS(13:0) | 0.001368 | Lipids:Glycerophospholipids | NA | 5 | 5.25 |
| PC(9:0) | 0.000442 | Lipids:Glycerophospholipids | NA | 5 | 5.27 |
| PC(15:0) | 0.008872 | Lipids:Glycerophospholipids | C00157 | 5 | -1.44 |
| PC (14:0) | 0.012489 | Lipids:Glycerophospholipids | C00157 | 7 | 1.19 |
| PC(14:0) | 0.021948 | Lipids:Glycerophospholipids | C00157 | 7 | 3.15 |
| PC(14:1) | 0.002947 | Lipids:Glycerophospholipids | C00157 | 7 | 4.99 |
| 1-tetradecanoyl-2-hexadecanoyl-sn-glycero-3-phosphoethanolamine | 0.004848 | Lipids:Glycerophospholipids | C00350 | 7 | 3.94 |
| PE(14:0) | 0.005746 | Lipids:Glycerophospholipids | C00350 | 7 | 5.10 |
| LysoPC(18:0) | 0.013036 | Lipids:Glycerophospholipids | C04230 | 5 | 1.89 |
| 9-mercaptodethiobiotin | 0.000796 | Metabolism of Cofactors and Vitamins | NA | 5 | -1.19 |
| Biotin | 0.000072 | Metabolism of Cofactors and Vitamins | C00120 | 8 | 1.14 |
| Nicotinamide | 1.9E-06 | Metabolism of Cofactors and Vitamins | C00153 | 6 | -5.74 |
| Nicotinate | 9.25E-07 | Metabolism of Cofactors and Vitamins | C00253 | 8 | -6.43 |
| Riboflavin | 8.29E-10 | Metabolism of Cofactors and Vitamins | C00255 | 6 | -1.28 |
| Dihydrobiopterin | 2.09E-07 | Metabolism of Cofactors and Vitamins | C00268 | 6 | -1.67 |
| Pyridoxine | 1.87E-10 | Metabolism of Cofactors and Vitamins | C00314 | 6 | -2.52 |
| Nicotinamide D-ribonucleotide | 2.76E-08 | Metabolism of Cofactors and Vitamins | C00455 | 6 | -8.38 |
| Pantetheine | 1.78E-08 | Metabolism of Cofactors and Vitamins | C00831 | 5 | -6.96 |
| Dephospho-CoA | 3.19E-06 | Metabolism of Cofactors and Vitamins | C00882 | 6 | -6.46 |
| 2,3-Dimethylmaleate | 0.00052 | Metabolism of Cofactors and Vitamins | C00922 | 5 | -1.04 |
| Porphobilinogen | 0.009061 | Metabolism of Cofactors and Vitamins | C00931 | 6 | -1.79 |
| 7,8-Diaminononanoate | 3.19E-05 | Metabolism of Cofactors and Vitamins | C01037 | 6 | -2.29 |
| Pantetheine 4'-phosphate | 6.85E-17 | Metabolism of Cofactors and Vitamins | C01134 | 6 | -7.26 |
| Nicotinate D-ribonucleotide | 1.46E-14 | Metabolism of Cofactors and Vitamins | C01185 | 6 | -5.39 |
| 2-Amino-4-hydroxy-6-hydroxymethyl-7,8-dihydropteridine | 1.06E-08 | Metabolism of Cofactors and Vitamins | C01300 | 8 | -3.00 |
| Dethiobiotin | 0.000467 | Metabolism of Cofactors and Vitamins | C01909 | 6 | -2.97 |
| N-Ribosylnicotinamide | 7.66E-11 | Metabolism of Cofactors and Vitamins | C03150 | 8 | -4.38 |
| 5-(2-Hydroxyethyl)-4-methylthiazole | 1.04E-12 | Metabolism of Cofactors and Vitamins | C042194 | 5 | -6.44 |
| 4-Methyl-5-(2-phosphoethyl)-thiazole | 0.000012 | Metabolism of Cofactors and Vitamins | C04327 | 6 | -4.85 |
| alpha-Ribazole | 3.13E-07 | Metabolism of Cofactors and Vitamins | C05775 | 6 | -2.09 |
| Nicotinate D-ribonucleoside | 4.52E-08 | Metabolism of Cofactors and Vitamins | C05841 | 5 | -3.83 |
| 2-Methyl-3-hydroxy-5-formylpyridine-4-carboxylate | 0.00396 | Metabolism of Cofactors and Vitamins | C06050 | 5 | -3.50 |
| UDP | 4.23E-15 | Nucleotide metabolism | C00015 | 6 | -7.73 |
| AMP | 2.68E-08 | Nucleotide metabolism | C00020 | 8 | -2.20 |
| GDP | 9.89E-05 | Nucleotide metabolism | C00035 | 8 | -1.82 |
| GTP | 1.61E-06 | Nucleotide metabolism | C00044 | 6 | -5.77 |
| CMP | 1.89E-15 | Nucleotide metabolism | C00055 | 6 | -5.97 |
| Uracil | 3.27E-06 | Nucleotide metabolism | C00106 | 8 | -1.42 |
| Adenine | 4E-08 | Nucleotide metabolism | C00147 | 6 | -2.53 |
| Thymine | 4.87E-07 | Nucleotide metabolism | C00178 | 6 | -2.13 |
| dADP | 1.68E-07 | Nucleotide metabolism | C00206 | 6 | -2.92 |
| Adenosine | 4.63E-08 | Nucleotide metabolism | C00212 | 6 | -2.71 |
| Thymidine | 2.28E-07 | Nucleotide metabolism | C00214 | 5 | -1.74 |
| Guanine | 1.88E-11 | Nucleotide metabolism | C00242 | 6 | -3.43 |
| Hypoxanthine | 2.16E-08 | Nucleotide metabolism | C00262 | 8 | -2.84 |
| dAMP | 2.76E-11 | Nucleotide metabolism | C00360 | 5 | -1.69 |
| Xanthine | 3.66E-14 | Nucleotide metabolism | C00385 | 8 | -6.30 |
| Cytidine | 5.13E-07 | Nucleotide metabolism | C00475 | 5 | -1.41 |
| Deoxyadenosine | 7.13E-07 | Nucleotide metabolism | C00559 | 6 | -1.42 |
| 3',5'-Cyclic AMP | 8.07E-06 | Nucleotide metabolism | C00575 | 8 | 3.77 |
| Xanthosine | 9.82E-11 | Nucleotide metabolism | C01762 | 8 | -6.33 |
| 7-Methyladenine | 7.96E-13 | Nucleotide metabolism | C02241 | 6 | -2.51 |
| Deoxyinosine | 0.021289 | Nucleotide metabolism | C05512 | 6 | -1.29 |
| L-gamma-glutamyl-L-isoleucine | 0.000128 | Peptide | NA | 7 | -1.99 |
| Gamma-Glutamyltyrosine | 0.004923 | Peptide | NA | 5 | -1.97 |
| Ser-Arg | 3.26E-05 | peptide(di) | NA | 7 | -6.66 |
| Lys-His | 5.35E-07 | peptide(di) | NA | 5 | -6.19 |
| Aspartyl-L-proline | 4.71E-06 | peptide(di) | NA | 5 | -6.16 |
| Asn-Pro | 2.5E-16 | peptide(di) | NA | 7 | -5.92 |
| Asp-Arg | 2.48E-07 | peptide(di) | NA | 7 | -4.11 |
| Ile-Ala | 1.61E-08 | peptide(di) | NA | 7 | -3.54 |
| Pro-His | 7.83E-07 | peptide(di) | NA | 5 | -2.49 |
| Trp-Gly | 0.005931 | peptide(di) | NA | 5 | -2.10 |
| Pro-Arg | 5.1E-06 | peptide(di) | NA | 5 | -1.80 |
| Glu-Thr | 0.029368 | peptide(di) | NA | 5 | -1.78 |
| Thr-Tyr | 2.89E-08 | peptide(di) | NA | 5 | -1.58 |
| Trp-Ser | 8.54E-07 | peptide(di) | NA | 5 | -1.52 |
| Val-Arg | 0.001315 | peptide(di) | NA | 7 | -1.09 |
| Thr-Arg | 0.019197 | peptide(di) | NA | 7 | -1.09 |
| Glycylproline | 4.56E-08 | peptide(di) | NA | 5 | -1.07 |
| Phe-Asp | 0.000107 | peptide(di) | NA | 5 | -1.07 |
| Ile-Tyr | 0.009318 | peptide(di) | NA | 5 | 3.50 |
| Glu-Glu | 0.000202 | peptide(di) | C01425 | 5 | -2.99 |
| L-Tyrosyl-L-arginine | 1.81E-07 | peptide(di) | C02993 | 7 | -2.55 |
| His-Leu | 3.78E-09 | Peptide(di) | C05010 | 7 | -1.87 |
| Ala-Ile-Ile-Pro | 3.18E-16 | peptide(tetra) | NA | 5 | -9.32 |
| His-Lys-Phe-His | 1.12E-05 | peptide(tetra) | NA | 5 | -9.25 |
| Lys-Thr-Ser-Tyr | 8.12E-16 | peptide(tetra) | NA | 7 | -8.97 |
| Asn-Leu-Val-Pro | 0.004333 | peptide(tetra) | NA | 7 | -8.92 |
| Ala-Gly-Pro-Tyr | 7.26E-24 | peptide(tetra) | NA | 5 | -8.88 |
| Arg-Cys-Pro-Arg | 6.09E-05 | peptide(tetra) | NA | 5 | -8.72 |
| Asp-Leu-Trp-Val | 1.45E-18 | peptide(tetra) | NA | 5 | -8.31 |
| Ile-Val-Pro-Pro | 2.53E-11 | peptide(tetra) | NA | 5 | -8.22 |
| Ala-Val-Pro-Pro | 3.81E-15 | peptide(tetra) | NA | 5 | -8.21 |
| Arg-Trp-Gln-Pro | 1.12E-15 | peptide(tetra) | NA | 5 | -8.06 |
| Asp-Trp-Gly-Gly | 1.38E-13 | peptide(tetra) | NA | 5 | -7.72 |
| Asp-Leu-Trp-Pro | 2.26E-15 | peptide(tetra) | NA | 5 | -7.57 |
| Ala-Gly-Pro-Pro | 9.3E-06 | peptide(tetra) | NA | 5 | -7.56 |
| Lys-Ser-Ser-Tyr | 1.12E-12 | peptide(tetra) | NA | 5 | -7.34 |
| Ala-Leu-Trp-Val | 6.78E-12 | peptide(tetra) | NA | 7 | -7.26 |
| Ala-Glu-Ile-Val | 0.004531 | peptide(tetra) | NA | 5 | -7.24 |
| Gln-Leu-Pro-Pro | 3.87E-14 | peptide(tetra) | NA | 5 | -7.00 |
| Asn-Asp-Gly-Pro | 6.59E-16 | peptide(tetra) | NA | 5 | -6.94 |
| Arg-Thr-Ser-Tyr | 0.007382 | peptide(tetra) | NA | 5 | -6.94 |
| Ala-Ala-Pro-Pro | 1.26E-13 | peptide(tetra) | NA | 5 | -6.91 |
| Glu-Phe-Pro-Pro | 1.09E-14 | peptide(tetra) | NA | 5 | -6.80 |
| His-Lys-Met-His | 3.39E-17 | peptide(tetra) | NA | 5 | -6.76 |
| Ala-Leu-Ser-His | 5.28E-16 | peptide(tetra) | NA | 5 | -6.71 |
| Ala-Ala-Asp-Pro | 9.72E-15 | peptide(tetra) | NA | 5 | -6.63 |
| Ala-Ser-Arg-Arg | 4.93E-16 | peptide(tetra) | NA | 7 | -6.52 |
| Arg-Met-Trp-Trp | 1.44E-15 | peptide(tetra) | NA | 5 | -6.45 |
| Lys-Thr-Thr-Thr | 1.64E-15 | peptide(tetra) | NA | 5 | -6.42 |
| Asp-Glu-Ile-Met | 1.21E-17 | peptide(tetra) | NA | 5 | -6.40 |
| Glu-Ala-Val | 1.5E-14 | peptide(tetra) | NA | 7 | -6.40 |
| Ala-Phe-Gln-Pro | 0.00217 | peptide(tetra) | NA | 5 | -6.37 |
| Ala-Gly-Pro-His | 0.000038 | peptide(tetra) | NA | 5 | -6.34 |
| Arg-Met-Cys-Pro | 9.72E-15 | peptide(tetra) | NA | 5 | -6.31 |
| Ala-Gly-Ser-Ser | 2.39E-05 | peptide(tetra) | NA | 5 | -6.22 |
| Ala-Lys-Phe-Gly | 2.18E-16 | peptide(tetra) | NA | 5 | -6.07 |
| Glu-Ile-Gln-Pro | 6.68E-16 | peptide(tetra) | NA | 5 | -5.91 |
| His-Gly-Pro-Pro | 1.53E-17 | peptide(tetra) | NA | 5 | -5.83 |
| Ala-Trp-Pro-Ser | 0.011709 | peptide(tetra) | NA | 7 | -5.82 |
| Ala-Leu-Ala-Ser | 4.08E-12 | peptide(tetra) | NA | 5 | -5.81 |
| Gln-Met-Phe-Tyr | 2.48E-14 | peptide(tetra) | NA | 5 | -5.80 |
| Glu-Ile-Ile-Pro | 6.59E-13 | peptide(tetra) | NA | 5 | -5.70 |
| Ala-Val-Gly-Pro | 1.98E-08 | peptide(tetra) | NA | 5 | -3.16 |
| Ala-Leu-Val-Asp | 0.000396 | peptide(tetra) | NA | 5 | -3.16 |
| Ala-Lys-Pro-Pro | 0.00057 | peptide(tetra) | NA | 5 | -3.14 |
| Ala-Leu-Gly-Pro | 1.02E-08 | peptide(tetra) | NA | 5 | -3.06 |
| Asp-Leu-Pro-Ser | 0.001167 | peptide(tetra) | NA | 5 | -3.02 |
| Gln-Leu-Lys-Pro | 2.57E-08 | peptide(tetra) | NA | 5 | -2.97 |
| Ile-Ile-Pro-Val | 1.28E-08 | peptide(tetra) | NA | 5 | -2.91 |
| Asn-Thr-Gln-Tyr | 0.00071 | peptide(tetra) | NA | 5 | -2.91 |
| Phe-Pro-Pro-Tyr | 1.31E-09 | peptide(tetra) | NA | 5 | -2.73 |
| Arg-Cys-Gly-Arg | 2.99E-07 | peptide(tetra) | NA | 5 | -2.70 |
| Ile-Ile-Pro-Pro | 2.53E-11 | peptide(tetra) | NA | 5 | -2.62 |
| Ala-Leu-Val-Ser | 5.52E-07 | peptide(tetra) | NA | 5 | -2.49 |
| Glu-Glu-Gln-Phe | 2.01E-09 | peptide(tetra) | NA | 5 | -2.49 |
| Ala-Glu-Glu-Pro | 1.3E-10 | peptide(tetra) | NA | 5 | -2.46 |
| Cys-Leu-Met-Trp | 1.75E-08 | peptide(tetra) | NA | 5 | -2.38 |
| Pro-Val-Val-Pro | 0.009605 | peptide(tetra) | NA | 5 | -2.38 |
| Arg-Leu-Thr-Ser | 0.009486 | peptide(tetra) | NA | 7 | -2.21 |
| Ala-Leu-Lys-Val | 9.26E-06 | peptide(tetra) | NA | 7 | -2.17 |
| Gln-Phe-Pro-Pro | 1.18E-06 | peptide(tetra) | NA | 5 | -2.07 |
| Ala-Leu-Thr-Pro | 3.5E-06 | peptide(tetra) | NA | 5 | -1.97 |
| Asn-Leu-Thr-Pro | 6.77E-07 | peptide(tetra) | NA | 5 | -1.97 |
| Asp-Glu-Ile-Trp | 0.001983 | peptide(tetra) | NA | 5 | -1.84 |
| Asp-Leu-Met-Pro | 0.001671 | peptide(tetra) | NA | 5 | -1.08 |
| Ala-Leu-Val-Pro | 0.009712 | peptide(tetra) | NA | 5 | -1.03 |
| Ala-Leu-Lys-Thr | 0.012204 | peptide(tetra) | NA | 7 | 1.89 |
| Ala-Met-Ala-Arg | 0.017862 | peptide(tetra) | NA | 5 | 2.21 |
| Gln-Phe-Pro-His | 4.84E-06 | peptide(tetra) | NA | 7 | 2.90 |
| Ala-Cys-Pro-Arg | 0.008964 | peptide(tetra) | NA | 5 | 2.99 |
| Ala-Thr-Gln-Pro | 0.008071 | peptide(tetra) | NA | 5 | 3.98 |
| Ala-Lys-Gly-Gly | 3.86E-16 | peptide(tetra) | NA | 7 | 5.37 |
| Lys-Phe-Pro | 1.36E-19 | peptide(tri) | NA | 5 | -8.41 |
| Phe-Phe-Pro | 1.41E-13 | peptide(tri) | NA | 5 | -8.20 |
| Glu-Pro-Pro | 9.06E-18 | peptide(tri) | NA | 5 | -8.18 |
| Ala-Pro-Ser | 3.69E-07 | Peptide(tri) | NA | 5 | -8.01 |
| Glu-Asp-His | 4.82E-14 | peptide(tri) | NA | 5 | -7.79 |
| Ile-Lys-Tyr | 0.003347 | Peptide(tri) | NA | 5 | -7.72 |
| Gly-Pro-Pro | 4.06E-19 | peptide(tri) | NA | 7 | -6.81 |
| Ile-Ile-Met | 0.000148 | Peptide(tri) | NA | 7 | -6.77 |
| Glu-Pro-Thr | 2.64E-15 | peptide(tri) | NA | 7 | -6.44 |
| Glu-Pro-Ser | 2.23E-15 | peptide(tri) | NA | 5 | -6.39 |
| Ala-Pro-Pro | 1.44E-18 | peptide(tri) | NA | 5 | -6.17 |
| Lys-Val-Tyr | 1.77E-15 | peptide(tri) | NA | 5 | -6.13 |
| Glu-Phe-Pro | 1.27E-17 | peptide(tri) | NA | 5 | -6.09 |
| Asp-Pro-Ser | 5.72E-15 | peptide(tri) | NA | 5 | -5.72 |
| Ile-Gly-His | 1.62E-14 | peptide(tri) | NA | 5 | -5.64 |
| Lys-Val-His | 0.000025 | Peptide(tri) | NA | 5 | -5.54 |
| Lys-Thr-Tyr | 0.020626 | Peptide(tri) | NA | 7 | -5.43 |
| Lys-Val-Pro | 0.000396 | Peptide(tri) | NA | 5 | -5.09 |
| Ile-Pro-Thr | 0.000243 | Peptide(tri) | NA | 5 | -4.64 |
| Lys-Val-Ser | 0.000466 | Peptide(tri) | NA | 5 | -4.40 |
| 2-hydroxy-4-methylthiobutanoate | 3.52E-17 | Undefined | NA | 7 | -9.01 |
| 2,3-dichloro-5-methyl-muconate | 5.51E-14 | Undefined | NA | 5 | -7.90 |
| N10-formyl-2_4-diaminodideazafolate | 3.33E-05 | Undefined | NA | 5 | -6.88 |
| phenylhydantoin | 9.98E-18 | Undefined | NA | 8 | -6.45 |
| Ophthalmicacid | 1.35E-16 | Undefined | NA | 5 | -6.25 |
| L-methioninamide | 2.87E-14 | Undefined | NA | 5 | -5.97 |
| beta-Citryl-L-glutamic acid | 0.006727 | Undefined | NA | 7 | -5.92 |
| Dihydrozeatin-9-N-glucoside-O-glucoside | 2.59E-18 | Undefined | NA | 5 | -5.75 |
| P-DPD | 8.17E-15 | Undefined | NA | 5 | -5.71 |
| 5-Methylcytidine | 1.16E-14 | Undefined | NA | 7 | -5.63 |
| L-Ala-&gamma;-D-Glu-Dap | 8.12E-16 | Undefined | NA | 6 | -5.63 |
| 3'-amino-3'-deoxyadenosine | 9.66E-05 | Undefined | NA | 7 | -5.29 |
| L-alpha-glutamyl-L-hydroxyproline | 0.000028 | Undefined | NA | 5 | -5.12 |
| N-acetyl-(L)-arginine | 1.7E-11 | Undefined | NA | 7 | -4.81 |
| 2-oxobut-3-enanoate | 8.95E-13 | Undefined | NA | 5 | -4.49 |
| (3a-5b)-24-oxo-24-[(2-sulfoethyl)amino]cholan-3-yl-b-D-Glucopyranosiduronicacid | 6.74E-05 | Undefined | NA | 5 | -4.40 |
| L-isoleucyl-L-proline | 0.004723 | Undefined | NA | 7 | -4.16 |
| 4-[N-&gamma;-L-glutamyl-)-p-(&beta;-aminoethyl)phenoxy-methyl]-2-(aminomethyl)furan | 0.000382 | Undefined | NA | 5 | -3.53 |
| D-galactosylononitol | 0.01208 | Undefined | NA | 7 | -3.26 |
| 1,6-anhydro-N-acetylmuramate | 0.000101 | Undefined | NA | 8 | -3.22 |
| N-Acetylcadaverine | 5.07E-08 | Undefined | NA | 7 | -3.20 |
| 6-Dimethylaminopurine | 0.001272 | Undefined | NA | 5 | -3.11 |
| &gamma;-thiomethyl glutamate | 2.77E-10 | Undefined | NA | 5 | -3.01 |
| glcNAc-1,6-anhMurNAc | 5.47E-07 | Undefined | NA | 8 | -2.90 |
| 19-oic-deoxycorticosterone | 3.26E-09 | Undefined | NA | 5 | -2.84 |
| dihydrothymidine | 2.02E-08 | Undefined | NA | 5 | -2.40 |
| 1-deoxyxylonojirimycin | 3.66E-05 | Undefined | NA | 7 | -2.30 |
| tetrahydropteroate | 8.24E-09 | Undefined | NA | 5 | -2.00 |
| N3-(4-methoxyfumaroyl)-L-2,3-diaminopropanoate | 0.015462 | Undefined | NA | 5 | -1.90 |
| N-Acetylglutamine | 2.94E-08 | Undefined | NA | 5 | -1.58 |
| N-acetyl -D- glucosaminitol | 2.86E-09 | Undefined | NA | 5 | -1.58 |
| 4-oxo-N-acetylneuraminate | 0.031472 | Undefined | NA | 7 | -1.42 |
| 8-Hydroxy-7-methylguanine | 3.15E-06 | Undefined | NA | 5 | -1.36 |
| 5-Methoxytryptophan | 3.12E-05 | Undefined | NA | 5 | -1.31 |
| Epsilon-(gamma-Glutamyl)-lysine | 0.000298 | Undefined | NA | 5 | -1.24 |
| Isovalerylglutamicacid | 4.47E-05 | Undefined | NA | 7 | -1.02 |
| beta-Carboline | 0.000183 | Undefined | NA | 5 | 2.05 |
| Palmiticamide | 0.004926 | Undefined | NA | 5 | 2.81 |
| 5-6-DihydroxyprostaglandinF1a | 0.006624 | Undefined | NA | 7 | 5.06 |
| Palmitoylglycerone phosphate | 5.29E-05 | Undefined | C01192 | 5 | -1.46 |
| N-(6-Aminohexanoyl)-6-aminohexanoate | 1.83E-06 | Undefined | C01255 | 7 | -1.58 |
| 2-Furoate | 5.24E-15 | Undefined | C01546 | 5 | -7.89 |
| Octylamine | 9.84E-07 | Undefined | C01740 | 7 | 1.70 |
| Nonanoyl-CoA | 0.000441 | Undefined | C01942 | 7 | -3.03 |
| 3-Methylguanine | 1.94E-10 | Undefined | C02230 | 6 | -3.44 |
| Xanthopterin-B2 | 0.000294 | Undefined | C02333 | 5 | 8.43 |
| 1-Methyladenosine | 2.53E-14 | Undefined | C02494 | 5 | -6.30 |
| N-Acetyl-L-leucine | 8.38E-10 | Undefined | C02710 | 7 | -3.68 |
| N-Acetylmethionine | 0.023634 | Undefined | C02712 | 5 | -1.93 |
| N-Acetylmuramate | 1.88E-08 | Undefined | C02713 | 8 | -1.18 |
| 5-Oxo-1,2-campholide | 0.015592 | Undefined | C02952 | 5 | 1.18 |
| 8-Oxodeoxycoformycin | 1.77E-07 | Undefined | C02957 | 7 | -1.10 |
| N-Acetyl-L-histidine | 1.3E-10 | Undefined | C02997 | 7 | -5.79 |
| Pyrimidine nucleoside | 7.34E-07 | Undefined | C03169 | 5 | -1.28 |
| D-2-Hydroxyisocaproate | 9.11E-10 | Undefined | C03264 | 7 | -6.12 |
| Glycerophosphoglycerol | 7.97E-12 | Undefined | C03274 | 7 | -6.83 |
| (Ac)2-L-Lys-D-Ala-D-Ala | 1.89E-05 | Undefined | C03326 | 5 | -1.26 |
| 2-Ethylhexyl phthalate | 0.000347 | Undefined | C03343 | 5 | 2.01 |
| N6,N6-Dimethyladenosine | 0.003755 | Undefined | C03416 | 5 | -2.23 |
| S-Acetylphosphopantetheine | 0.009053 | Undefined | C03725 | 5 | -3.65 |
| Ethyl (R)-3-hydroxyhexanoate | 0.001348 | Undefined | C03864 | 5 | 1.10 |
| 1-Oleoylglycerophosphocholine | 0.021189 | Undefined | C03916 | 5 | 1.65 |
| N-Hydroxy-2-acetamidofluorene | 0.005263 | Undefined | C03954 | 5 | -2.40 |
| 1-Linoleoylglycerophosphocholine | 0.016923 | Undefined | C04100 | 5 | 1.72 |
| 1-Deoxy-D-altro-heptulose 7-phosphate | 7.07E-11 | Undefined | C04359 | 5 | -6.98 |
| 2,7-Anhydro-alpha-N-acetylneuraminic acid | 1.82E-10 | Undefined | C04521 | 5 | -2.37 |
| Isoquinoline | 0.00121 | Undefined | C06323 | 6 | -1.64 |
| 5'-Butyrylphosphouridine | 1.52E-15 | Undefined | C06436 | 5 | 7.84 |
| cis-1,2-Dihydroxy-1,2-dihydrodibenzothiophene | 0.000259 | Undefined | C06721 | 5 | -2.11 |
| L-gamma-Glutamyl-L-hypoglycin | 0.000978 | Undefined | C08280 | 6 | -3.27 |
| L-Hypoglycin | 0.000135 | Undefined | C08287 | 8 | 2.10 |
| 8-(3,3-Dimethylallyl)spatheliachromene | 1.88E-06 | Undefined | C09002 | 5 | -1.31 |
| Autumnolide | 0.001456 | Undefined | C09346 | 5 | 8.54 |
| alpha-Irone | 0.002703 | Undefined | C09690 | 7 | 3.88 |
| Deoxymannojirimycin | 1.5E-09 | Undefined | C10141 | 7 | -5.47 |
| N-Acetyl-leucyl-leucine | 0.0005 | Undefined | C11333 | 7 | -4.07 |
| 5'-Dehydroadenosine | 1.14E-05 | Undefined | C11500 | 5 | -3.40 |
| Succinyl proline | 0.00156 | Undefined | C11711 | 5 | -2.45 |
| Cyclic ADP-ribose | 5.18E-10 | Undefined | C13050 | 5 | -4.89 |
| (S)-ATPA | 2.03E-05 | Undefined | C13733 | 7 | -1.24 |
| Di(2-ethylhexyl) adipate | 0.013884 | Undefined | C14240 | 5 | 3.19 |
| Tributyl phosphate | 0.016219 | Undefined | C14439 | 5 | 2.92 |
| (R)-4'-Deoxyindenestrol | 3.63E-06 | Undefined | C14541 | 7 | -6.96 |
| 1-(beta-D-Ribofuranosyl)-1,4-dihydronicotinamide | 1.59E-07 | Undefined | C15497 | 8 | -8.02 |
| Mycinamicin VI | 0.000473 | Undefined | C15682 | 5 | -5.25 |
| allylcysteine | 7.34E-19 | Undefined | C16759 | 5 | -8.45 |
| **4 hour** | | | | | |
| **Putative Metabolite** | ***p.* value** | **Map** | **Kegg ID** | **Confidence** | **log2(FC)** |
| L-Glutamate | 5.21E-06 | Amino acid metabolism | C00025 | 8 | -2.57 |
| L-Alanine | 1.64E-06 | Amino acid metabolism | C00041 | 6 | -2.46 |
| L-Lysine | 3.91E-11 | Amino acid metabolism | C00047 | 8 | -4.30 |
| Glutathione | 1.08E-10 | Amino acid metabolism | C00051 | 8 | -6.31 |
| L-Arginine | 6.62E-06 | Amino acid metabolism | C00062 | 8 | -2.03 |
| L-Glutamine | 5.59E-09 | Amino acid metabolism | C00064 | 6 | -4.81 |
| L-Serine | 4.21E-05 | Amino acid metabolism | C00065 | 8 | -1.05 |
| L-Methionine | 3.32E-07 | Amino acid metabolism | C00073 | 8 | -2.42 |
| L-Ornithine | 3.02E-08 | Amino acid metabolism | C00077 | 8 | -3.86 |
| L-Tryptophan | 9.87E-07 | Amino acid metabolism | C00078 | 6 | -2.24 |
| L-Phenylalanine | 2.53E-06 | Amino acid metabolism | C00079 | 8 | -2.01 |
| L-Tyrosine | 2.42E-07 | Amino acid metabolism | C00082 | 8 | -2.75 |
| Choline | 0.002195 | Amino acid metabolism | C00114 | 8 | -1.08 |
| L-Leucine | 5.71E-07 | Amino acid metabolism | C00123 | 8 | -2.31 |
| L-Histidine | 0.000173 | Amino acid metabolism | C00135 | 6 | -1.82 |
| 3-Methyl-2-oxobutanoic acid | 5.74E-05 | Amino acid metabolism | C00141 | 6 | -2.37 |
| L-Proline | 9.39E-07 | Amino acid metabolism | C00148 | 8 | -2.33 |
| Phenylpyruvate | 1.48E-07 | Amino acid metabolism | C00166 | 8 | -2.86 |
| Carbamoyl phosphate | 0.001652 | Amino acid metabolism | C00169 | 8 | 2.05 |
| 5'-Methylthioadenosine | 1.26E-09 | Amino acid metabolism | C00170 | 6 | -4.31 |
| L-Valine | 6.48E-08 | Amino acid metabolism | C00183 | 8 | -3.36 |
| L-Threonine | 0.000305 | Amino acid metabolism | C00188 | 6 | -1.28 |
| 4-Methyl-2-oxopentanoate | 9.72E-06 | Amino acid metabolism | C00233 | 6 | -3.73 |
| L-Citrulline | 6.96E-16 | Amino acid metabolism | C00327 | 6 | -11.69 |
| L-Kynurenine | 0.028586 | Amino acid metabolism | C00328 | 6 | -1.85 |
| Indolepyruvate | 2.67E-08 | Amino acid metabolism | C00331 | 6 | -3.72 |
| 4-Aminobutanoate | 2.81E-10 | Amino acid metabolism | C00334 | 8 | -3.58 |
| 3,4-Dihydroxy-L-phenylalanine | 0.000396 | Amino acid metabolism | C00355 | 7 | -2.54 |
| N-Acetylornithine | 0.000294 | Amino acid metabolism | C00437 | 6 | -4.78 |
| N-Formimino-L-glutamate | 6.27E-08 | Amino acid metabolism | C00439 | 8 | -6.80 |
| N6-(L-1,3-Dicarboxypropyl)-L-lysine | 0.016233 | Amino acid metabolism | C00449 | 5 | 2.32 |
| L-Carnitine | 4.73E-06 | Amino acid metabolism | C00487 | 5 | -1.16 |
| N-Acetyl-L-glutamate | 7.78E-08 | Amino acid metabolism | C00624 | 8 | -4.05 |
| 5-Hydroxy-L-tryptophan | 9.74E-08 | Amino acid metabolism | C00643 | 5 | -2.61 |
| gamma-L-Glutamyl-L-cysteine | 0.000279 | Amino acid metabolism | C00669 | 6 | -4.14 |
| Urocanate | 4.27E-08 | Amino acid metabolism | C00785 | 8 | -3.86 |
| Pantothenate | 4.55E-07 | Amino acid metabolism | C00864 | 6 | -5.73 |
| L-2-Aminoadipate | 8.84E-10 | Amino acid metabolism | C00956 | 8 | -3.09 |
| 5-Aminopentanamide | 2.11E-07 | Amino acid metabolism | C00990 | 5 | -5.18 |
| D-Alanyl-D-alanine | 1.19E-07 | Amino acid metabolism | C00993 | 6 | -4.82 |
| 4-Guanidinobutanoate | 7.4E-08 | Amino acid metabolism | C01035 | 8 | -4.55 |
| N-Formyl-L-glutamate | 1.93E-05 | Amino acid metabolism | C01045 | 6 | -7.13 |
| UDP-N-acetylmuramate | 1.06E-14 | Amino acid metabolism | C01050 | 6 | -7.03 |
| O-Succinyl-L-homoserine | 7.85E-11 | Amino acid metabolism | C01118 | 6 | -4.67 |
| N(pi)-Methyl-L-histidine | 0.02258 | Amino acid metabolism | C01152 | 5 | -1.09 |
| 3-(4-Hydroxyphenyl)pyruvate | 9.78E-09 | Amino acid metabolism | C01179 | 8 | -3.64 |
| 3-(2-Hydroxyphenyl)propanoate | 7.5E-11 | Amino acid metabolism | C01198 | 5 | -7.28 |
| 1-Aminocyclopropane-1-carboxylate | 1.21E-07 | Amino acid metabolism | C01234 | 8 | -2.97 |
| N-Acetyl-L-glutamate 5-semialdehyde | 1.84E-07 | Amino acid metabolism | C01250 | 6 | -2.19 |
| 4-(2-Aminophenyl)-2,4-dioxobutanoate | 0.000237 | Amino acid metabolism | C01252 | 6 | -2.12 |
| 4-Oxoproline | 0.000144 | Amino acid metabolism | C01877 | 7 | -1.09 |
| L-Histidinal | 1.41E-06 | Amino acid metabolism | C01929 | 6 | -1.48 |
| Indolelactate | 2.07E-10 | Amino acid metabolism | C02043 | 7 | -9.21 |
| O-Acetylcarnitine | 4.74E-05 | Amino acid metabolism | C02571 | 5 | -2.20 |
| L-Formylkynurenine | 2.25E-06 | Amino acid metabolism | C02700 | 6 | -1.81 |
| N-Acetylputrescine | 1.32E-12 | Amino acid metabolism | C02714 | 6 | -7.50 |
| N6-Acetyl-L-lysine | 1.84E-08 | Amino acid metabolism | C02727 | 7 | -3.37 |
| N6-Methyl-L-lysine | 1.83E-08 | Amino acid metabolism | C02728 | 7 | -3.08 |
| L-Lysine 1,6-lactam | 4.4E-09 | Amino acid metabolism | C02837 | 7 | -2.39 |
| 4-Acetamidobutanoate | 1.73E-10 | Amino acid metabolism | C02946 | 8 | -2.78 |
| 5-Acetamidopentanoate | 1.01E-05 | Amino acid metabolism | C03087 | 5 | -2.45 |
| N-Formyl-L-methionine | 6.83E-19 | Amino acid metabolism | C03145 | 5 | -8.52 |
| Imidazol-5-yl-pyruvate | 1.73E-08 | Amino acid metabolism | C03277 | 5 | -4.04 |
| N2-Succinyl-L-arginine | 1.37E-10 | Amino acid metabolism | C03296 | 8 | -13.92 |
| N-(L-Arginino)succinate | 2.01E-15 | Amino acid metabolism | C03406 | 6 | -7.67 |
| N2-Succinyl-L-ornithine | 2.63E-07 | Amino acid metabolism | C03415 | 6 | -6.07 |
| N-Acetyl-L-phenylalanine | 0.000344 | Amino acid metabolism | C03519 | 5 | -6.06 |
| 4-Imidazolone-5-propanoate | 1.15E-05 | Amino acid metabolism | C03680 | 6 | -9.10 |
| 5-Guanidino-2-oxopentanoate | 2.03E-08 | Amino acid metabolism | C03771 | 5 | -2.93 |
| (S)-1-Pyrroline-5-carboxylate | 0.000133 | Amino acid metabolism | C03912 | 6 | -1.71 |
| N6-Acetyl-N6-hydroxy-L-lysine | 6.19E-07 | Amino acid metabolism | C03955 | 7 | -5.39 |
| N2-(D-1-Carboxyethyl)-L-lysine.2 | 0.000019 | Amino acid metabolism | C04020 | 7 | -7.85 |
| N2-(D-1-Carboxyethyl)-L-arginine | 0.028499 | Amino acid metabolism | C04137 | 5 | -1.19 |
| alpha-N-Phenylacetyl-L-glutamine | 0.000039 | Amino acid metabolism | C04148 | 5 | -1.39 |
| UDP-N-acetylmuramoyl-L-alanyl-D-glutamyl-6-carboxy-L-lysyl-D-alanyl- D-alanine | 1.07E-06 | Amino acid metabolism | C04882 | 8 | -5.59 |
| (1+100:187-Ribosylimidazole)-4-acetate | 0.003098 | Amino acid metabolism | C05131 | 5 | -2.92 |
| 4-Imidazolone-5-acetate | 0.000391 | Amino acid metabolism | C05133 | 5 | -4.28 |
| Hydantoin-5-propionate | 1.56E-15 | Amino acid metabolism | C05565 | 5 | -8.67 |
| Hercynine | 2.7E-08 | Amino acid metabolism | C05575 | 5 | -2.65 |
| 3,4-Dihydroxymandelate | 0.000121 | Amino acid metabolism | C05580 | 6 | -4.88 |
| 3-Methoxy-4-hydroxyphenylglycolaldehyde | 1.25E-11 | Amino acid metabolism | C05583 | 6 | -8.63 |
| N-Succinyl-L-glutamate | 8.04E-06 | Amino acid metabolism | C05931 | 8 | -6.53 |
| (S)-2-Aceto-2-hydroxybutanoate | 7.97E-10 | Amino acid metabolism | C06006 | 8 | -4.96 |
| gamma-Glutamyl-beta-aminopropiononitrile | 1.37E-05 | Amino acid metabolism | C06114 | 5 | -1.92 |
| Hydroxymethylphosphonate | 2.89E-05 | Amino acid metabolism | C06455 | 7 | -6.05 |
| 3-(Methylthio)propionic acid | 2.88E-05 | Amino acid metabolism | C08276 | 6 | -3.86 |
| 1,2-Dihydroxy-5-(methylthio)pent-1-en-3-one | 2.86E-05 | Amino acid metabolism | C15606 | 6 | -4.20 |
| gamma-Glutamyl-gamma-aminobutyraldehyde | 1.21E-07 | Amino acid metabolism | C15700 | 5 | -2.48 |
| Sinapate | 7.96E-08 | Biosynthesis of Secondary metabolite | C00482 | 6 | -6.69 |
| dTDP-6-deoxy-L-mannose | 1.12E-11 | Biosynthesis of Secondary metabolite | C03319 | 6 | -6.44 |
| 7-O-Acetylsalutaridinol | 0.001864 | Biosynthesis of Secondary metabolite | C05322 | 5 | -3.81 |
| Deoxyguanidinoproclavaminic acid | 1.49E-08 | Biosynthesis of Secondary metabolite | C06656 | 6 | -4.22 |
| Proclavaminic acid | 4.75E-06 | Biosynthesis of Secondary metabolite | C06658 | 6 | -7.73 |
| Dihydroclavaminic acid | 0.000186 | Biosynthesis of Secondary metabolite | C06659 | 6 | 1.34 |
| 5-Acetylamino-6-formylamino-3-methyluracil | 1.46E-17 | Biosynthesis of Secondary metabolite | C16365 | 7 | 7.38 |
| CoA | 4.76E-12 | Carbohydrate metabolism | C00010 | 8 | -6.09 |
| CoA.1 | 1.38E-12 | Carbohydrate metabolism | C00010 | 8 | -7.70 |
| Pyruvate | 2.36E-11 | Carbohydrate metabolism | C00022 | 6 | -4.43 |
| Acetyl-CoA | 1.47E-07 | Carbohydrate metabolism | C00024 | 8 | -4.59 |
| UDP-glucose | 5.12E-15 | carbohydrate metabolism | C00029 | 6 | -10.89 |
| Succinate | 3.06E-11 | Carbohydrate metabolism | C00042 | 8 | -6.95 |
| UDP-N-acetyl-D-glucosamine | 2.79E-16 | Carbohydrate metabolism | C00043 | 6 | -6.43 |
| Phosphoenolpyruvate | 9.9E-09 | Carbohydrate metabolism | C00074 | 8 | -3.76 |
| Succinyl-CoA | 6.89E-14 | Carbohydrate metabolism | C00091 | 6 | -7.71 |
| D-Fructose | 1.96E-08 | Carbohydrate metabolism | C00095 | 6 | -3.88 |
| D-Ribose | 0.000224 | Carbohydrate metabolism | C00121 | 8 | -2.13 |
| (S)-Malate | 5.7E-08 | Carbohydrate metabolism | C00149 | 8 | -4.40 |
| Citrate | 0.002188 | Carbohydrate metabolism | C00158 | 8 | -1.72 |
| UDP-glucuronate | 5.16E-08 | Carbohydrate metabolism | C00167 | 8 | -8.22 |
| S-Lactate | 5.9E-07 | Carbohydrate metabolism | C00186 | 6 | -3.05 |
| 3-Phospho-D-glycerate | 2.65E-07 | Carbohydrate metabolism | C00197 | 8 | -5.39 |
| D-Glucono-1,5-lactone | 6.09E-09 | Carbohydrate metabolism | C00198 | 6 | -2.94 |
| D-Glycerate | 6.31E-10 | Carbohydrate metabolism | C00258 | 6 | -6.80 |
| N-Acetylneuraminate | 0.001056 | Carbohydrate metabolism | C00270 | 6 | -7.58 |
| D-Erythrose 4-phosphate | 1.13E-09 | Carbohydrate metabolism | C00279 | 6 | -3.61 |
| D-Glucosamine | 2.32E-09 | Carbohydrate metabolism | C00329 | 5 | -7.77 |
| 6-Phospho-D-gluconate | 5.59E-05 | Carbohydrate metabolism | C00345 | 8 | -6.24 |
| Mannitol | 2.71E-07 | Carbohydrate metabolism | C00392 | 6 | -5.64 |
| D-Xylonate | 0.013055 | Carbohydrate metabolism | C00502 | 5 | 1.16 |
| L-Arabitol | 7E-10 | Carbohydrate metabolism | C00532 | 6 | -3.45 |
| D-Mannitol 1-phosphate | 1.37E-20 | Carbohydrate metabolism | C00644 | 7 | -6.54 |
| 2-Deoxy-D-ribose 1-phosphate | 6.62E-07 | Carbohydrate metabolism | C00672 | 8 | -5.62 |
| Diacetyl | 8.37E-05 | Carbohydrate metabolism | C00741 | 6 | -5.48 |
| R,R-Tartaric acid.6 | 0.000528 | Carbohydrate metabolism | C00898 | 6 | -1.87 |
| 2-Acetolactate | 3.28E-07 | Carbohydrate metabolism | C00900 | 6 | -2.05 |
| 6-Phospho-2-dehydro-D-gluconate | 1.75E-05 | Carbohydrate metabolism | C01218 | 6 | -2.56 |
| FA trihydroxy(4:0) | 0.000644 | Carbohydrate metabolism | C01620 | 8 | -1.86 |
| 3-Ethylmalate | 0.000015 | Carbohydrate metabolism | C01989 | 7 | -2.19 |
| 2-Methylcitrate | 9.19E-08 | Carbohydrate metabolism | C02225 | 8 | -3.72 |
| (R)-S-Lactoylglutathione | 7.27E-07 | Carbohydrate metabolism | C03451 | 6 | -5.00 |
| 1-O-Methyl-myo-inositol | 2.94E-11 | Carbohydrate metabolism | C03659 | 7 | -3.85 |
| 2-(alpha-Hydroxyethyl)thiamine diphosphate | 0.023746 | Carbohydrate metabolism | C05125 | 6 | -2.57 |
| D-Sedoheptulose 7-phosphate | 2.6E-15 | Carbohydrate metabolism | C05382 | 6 | -9.15 |
| dTDP-4-acetamido-4,6-dideoxy-D-glucose | 2.78E-07 | Carbohydrate metabolism | C06018 | 5 | -8.54 |
| ATP | 6.21E-13 | Energy Metabolism | C00002 | 6 | -8.13 |
| NAD | 1.52E-11 | Energy Metabolism | C00003 | 8 | -6.76 |
| NADP | 1.41E-06 | Energy Metabolism | C00006 | 6 | -4.76 |
| Orthophosphate | 0.000801 | Energy Metabolism | C00009 | 7 | -4.24 |
| D-Fructose 1,6-bisphosphate | 6.86E-08 | Energy Metabolism | C00354 | 6 | -3.96 |
| Sedoheptulose | 1.86E-08 | Energy Metabolism | C02076 | 5 | -5.55 |
| 9-methoxy-heptadecanoic acid | 0.007042 | Fatty Acyls[FA] | NA | 5 | 1.27 |
| 3-Deoxy-D-manno-octulosonate | 7.33E-10 | Glycan Biosynthesis and Metabolism | C01187 | 6 | -7.57 |
| UDP-3-O-(3-hydroxytetradecanoyl)-N-acetylglucosamine | 0.000404 | Glycan Biosynthesis and Metabolism | C04738 | 6 | -5.38 |
| 2,3-Bis(3-hydroxytetradecanoyl)-beta-D-glucosaminyl 1-phosphate | 2.96E-06 | Glycan Biosynthesis and Metabolism | C04824 | 6 | -3.46 |
| 1-pentadecanoyl-2-(9Z-hexadecenoyl)-sn-glycero-3-phosphocholine | 0.005703 | Glycerophospholipids[GP] | NA | 5 | 2.72 |
| 1-tetradecanoyl-2-(9Z-octadecenoyl)-sn-glycero-3-phosphocholine | 0.001937 | Glycerophospholipids[GP] | NA | 5 | -1.27 |
| Hexadecanoic acid | 0.009167 | Lipid Metabolism | C00249 | 8 | 1.71 |
| LPA(16:0) | 0.000466 | Lipid Metabolism | C00416 | 7 | -1.14 |
| Choline phosphate | 3.44E-07 | Lipid Metabolism | C00588 | 6 | -2.40 |
| sn-glycero-3-Phosphocholine | 0.016321 | Lipid Metabolism | C00670 | 6 | 1.06 |
| Tetradecanoic acid | 0.003902 | Lipid Metabolism | C06424 | 5 | 1.40 |
| (9Z)-Hexadecenoic acid | 0.001601 | Lipid Metabolism | C08362 | 5 | 2.58 |
| 2-C-Methyl-D-erythritol 4-phosphate | 2.69E-06 | Lipid Metabolism | C11434 | 6 | -3.18 |
| 2-C-Methyl-D-erythritol 4-phosphate.1 | 4.85E-07 | Lipid Metabolism | C11434 | 6 | -3.53 |
| FA (17:0) | 0.013295 | Lipids:Fatty Acyls | NA | 5 | 1.49 |
| FA oxo(16:0) | 0.008561 | Lipids:Fatty Acyls | NA | 5 | 1.02 |
| FA (20:4) | 0.006326 | Lipids:Fatty Acyls | C00219 | 6 | 1.33 |
| FA (18:1) | 0.009598 | Lipids:Fatty Acyls | C00712 | 6 | 1.80 |
| Lipoate | 6.63E-16 | Lipids:Fatty Acyls | C00725 | 6 | 8.53 |
| Dodecylaldehyde | 0.00049 | Lipids:Fatty Acyls | C02278 | 7 | -4.59 |
| 5-Hydroxypentanoate | 8.7E-14 | Lipids:Fatty Acyls | C02804 | 5 | -6.15 |
| O-Butanoylcarnitine | 0.000545 | Lipids:Fatty Acyls | C02862 | 5 | -4.23 |
| FA (18:3) | 0.004732 | Lipids:Fatty Acyls | C06426 | 6 | 1.74 |
| FA hydroxy(4:0) | 0.000151 | Lipids:Fatty Acyls | C11842 | 6 | 1.11 |
| 2S-Hydroxytetradecanoic acid | 0.015563 | Lipids:Fatty Acyls | C13790 | 7 | -1.15 |
| FA (20:0) | 0.008833 | Lipids:Fatty Acyls | C16526 | 5 | 2.42 |
| Pentadecanoic acid | 0.004404 | Lipids:Fatty Acyls | C16537 | 5 | 1.42 |
| LysoPE(16:1) | 1.07E-05 | Lipids:Glycerophospholipid | NA | 7 | 2.90 |
| PE(O-20:0/22:4(7Z,10Z,13Z,16Z)) | 5.74E-07 | Lipids:Glycerophospholipid | NA | 5 | -3.86 |
| PG(15:0) | 0.024754 | Lipids:Glycerophospholipids | NA | 5 | -2.67 |
| LysoPE(18:1) | 0.02182 | Lipids:Glycerophospholipids | NA | 5 | 1.02 |
| PGP(16:0) | 0.015294 | Lipids:Glycerophospholipids | NA | 7 | -1.26 |
| PA(19:1) | 0.009276 | Lipids:Glycerophospholipids | NA | 5 | 1.80 |
| PS (18:1) | 0.008953 | Lipids:Glycerophospholipids | NA | 7 | -1.91 |
| 1-decanoyl-2-tridecanoyl-sn-glycero-3-phosphocholine | 0.007268 | Lipids:Glycerophospholipids | NA | 5 | 2.24 |
| PG(13:0) | 0.007026 | Lipids:Glycerophospholipids | NA | 5 | 1.38 |
| PG(16:0) | 0.002967 | Lipids:Glycerophospholipids | NA | 7 | 1.81 |
| PS(13:0) | 0.001368 | Lipids:Glycerophospholipids | NA | 5 | 5.63 |
| LysoPE(14:0) | 0.001256 | Lipids:Glycerophospholipids | NA | 5 | 1.36 |
| PG (14:0) | 0.00045 | Lipids:Glycerophospholipids | NA | 5 | 1.71 |
| PC(9:0) | 0.000442 | Lipids:Glycerophospholipids | NA | 5 | 6.45 |
| PG(16:1) | 0.00016 | Lipids:Glycerophospholipids | NA | 7 | 1.77 |
| LysoPE(14:1) | 0.00013 | Lipids:Glycerophospholipids | NA | 5 | 6.73 |
| 1,2-dihexadecanoyl-sn-glycero-3-cytidine-5'-diphosphate | 2.09E-12 | Lipids:Glycerophospholipids | NA | 5 | -10.08 |
| PC(15:0) | 0.008872 | Lipids:Glycerophospholipids | C00157 | 5 | -2.78 |
| PC(14:1) | 0.005114 | Lipids:Glycerophospholipids | C00157 | 7 | 4.43 |
| PE(14:0) | 0.019928 | Lipids:Glycerophospholipids | C00350 | 5 | 2.27 |
| PE (14:0/16:0) | 0.006168 | Lipids:Glycerophospholipids | C00350 | 7 | 1.15 |
| PC (14:1) | 0.003341 | Lipids:Glycerophospholipids | C04230 | 5 | 1.19 |
| FAD | 0.00056 | Metabolism of Cofactors and Vitamins | C00016 | 8 | -1.06 |
| Biotin | 0.000072 | Metabolism of Cofactors and Vitamins | C00120 | 8 | 5.34 |
| Nicotinamide | 9.87E-11 | Metabolism of Cofactors and Vitamins | C00153 | 8 | -3.86 |
| Nicotinate | 9.25E-07 | Metabolism of Cofactors and Vitamins | C00253 | 8 | -5.29 |
| Riboflavin | 8.29E-10 | Metabolism of Cofactors and Vitamins | C00255 | 6 | -1.14 |
| Dihydrobiopterin | 2.09E-07 | Metabolism of Cofactors and Vitamins | C00268 | 6 | -1.38 |
| Pyridoxine | 1.87E-10 | Metabolism of Cofactors and Vitamins | C00314 | 6 | -3.47 |
| Nicotinamide D-ribonucleotide | 2.76E-08 | Metabolism of Cofactors and Vitamins | C00455 | 6 | -7.72 |
| Pantetheine | 1.78E-08 | Metabolism of Cofactors and Vitamins | C00831 | 5 | -6.76 |
| 4-Pyridoxate | 5.53E-08 | Metabolism of Cofactors and Vitamins | C00847 | 5 | -1.84 |
| Dephospho-CoA | 3.34E-11 | Metabolism of Cofactors and Vitamins | C00882 | 6 | -7.95 |
| 2,3-Dimethylmaleate | 0.00052 | Metabolism of Cofactors and Vitamins | C00922 | 5 | -1.56 |
| 7,8-Diaminononanoate | 3.19E-05 | Metabolism of Cofactors and Vitamins | C01037 | 6 | -4.47 |
| 8-Amino-7-oxononanoate | 0.018128 | Metabolism of Cofactors and Vitamins | C01092 | 6 | -1.11 |
| Pantetheine 4'-phosphate | 6.85E-17 | Metabolism of Cofactors and Vitamins | C01134 | 6 | -7.99 |
| Nicotinate D-ribonucleotide | 1.46E-14 | Metabolism of Cofactors and Vitamins | C01185 | 6 | -6.21 |
| 2-Amino-4-hydroxy-6-hydroxymethyl-7,8-dihydropteridine | 1.06E-08 | Metabolism of Cofactors and Vitamins | C01300 | 8 | -4.22 |
| Dethiobiotin | 1.23E-06 | Metabolism of Cofactors and Vitamins | C01909 | 6 | -2.10 |
| N-Ribosylnicotinamide | 7.66E-11 | Metabolism of Cofactors and Vitamins | C03150 | 8 | -4.48 |
| 5-(2-Hydroxyethyl)-4-methylthiazole | 1.04E-12 | Metabolism of Cofactors and Vitamins | C042194 | 5 | -8.42 |
| 4-Methyl-5-(2-phosphoethyl)-thiazole | 0.000012 | Metabolism of Cofactors and Vitamins | C04327 | 6 | -7.70 |
| Nicotianamine | 1.17E-11 | Metabolism of Cofactors and Vitamins | C05324 | 7 | -4.86 |
| alpha-Ribazole | 4.21E-09 | Metabolism of Cofactors and Vitamins | C05775 | 8 | -2.19 |
| Nicotinate D-ribonucleoside | 4.52E-08 | Metabolism of Cofactors and Vitamins | C05841 | 5 | -3.98 |
| UDP | 4.23E-15 | Nucleotide metabolism | C00015 | 6 | -9.23 |
| AMP | 2.68E-08 | Nucleotide metabolism | C00020 | 8 | -2.88 |
| GDP | 9.89E-05 | Nucleotide metabolism | C00035 | 8 | -2.17 |
| GTP | 1.61E-06 | Nucleotide metabolism | C00044 | 6 | -5.16 |
| CMP | 1.89E-15 | Nucleotide metabolism | C00055 | 6 | -7.53 |
| UTP | 6.74E-09 | Nucleotide metabolism | C00075 | 6 | -6.86 |
| UMP | 3.69E-14 | Nucleotide metabolism | C00105 | 6 | -7.03 |
| Uracil | 3.27E-06 | Nucleotide metabolism | C00106 | 8 | -1.44 |
| GMP | 0.002165 | Nucleotide metabolism | C00144 | 8 | -6.07 |
| Adenine | 2E-10 | Nucleotide metabolism | C00147 | 6 | -3.66 |
| Thymine | 1.88E-06 | Nucleotide metabolism | C00178 | 6 | -1.95 |
| dADP | 1.68E-07 | Nucleotide metabolism | C00206 | 6 | -3.23 |
| Adenosine | 9.87E-12 | Nucleotide metabolism | C00212 | 6 | -6.03 |
| Guanine | 1.88E-11 | Nucleotide metabolism | C00242 | 6 | -4.60 |
| Hypoxanthine | 2.16E-08 | Nucleotide metabolism | C00262 | 8 | -2.72 |
| dAMP | 2.76E-11 | Nucleotide metabolism | C00360 | 5 | -2.94 |
| dTDP | 4.93E-06 | Nucleotide metabolism | C00363 | 6 | -3.67 |
| Cytosine | 1.39E-05 | Nucleotide metabolism | C00380 | 6 | -1.84 |
| Xanthine | 3.66E-14 | Nucleotide metabolism | C00385 | 8 | -7.70 |
| Guanosine | 5.33E-05 | Nucleotide metabolism | C00387 | 6 | -1.37 |
| Cytidine | 5.13E-07 | Nucleotide metabolism | C00475 | 5 | -3.06 |
| Deoxyadenosine | 9.99E-07 | Nucleotide metabolism | C00559 | 6 | -1.91 |
| 3',5'-Cyclic AMP | 8.07E-06 | Nucleotide metabolism | C00575 | 8 | 8.10 |
| Xanthosine 5'-phosphate | 7.42E-06 | Nucleotide metabolism | C00655 | 6 | -7.10 |
| dCDP | 3.42E-09 | Nucleotide metabolism | C00705 | 6 | -6.64 |
| Xanthosine | 9.82E-11 | Nucleotide metabolism | C01762 | 8 | -9.40 |
| 7-Methyladenine | 7.96E-13 | Nucleotide metabolism | C02241 | 6 | -1.90 |
| Deoxyinosine | 0.021289 | Nucleotide metabolism | C05512 | 6 | -2.55 |
| L-gamma-glutamyl-L-isoleucine | 0.012521 | Peptide | NA | 5 | -3.16 |
| Gamma-Glutamyltyrosine | 0.004923 | Peptide | NA | 5 | -3.18 |
| L-beta-aspartyl-L-leucine | 2.34E-07 | Peptide | NA | 7 | -3.47 |
| gamma-L-Glutamyl-D-alanine | 0.000513 | Peptide | C03738 | 7 | -4.31 |
| Gamma-Glutamylglutamine | 1.97E-10 | Peptide | C05283 | 5 | -3.74 |
| Ile-Tyr | 0.009318 | peptide(di) | NA | 5 | 3.32 |
| Trp-Gly | 0.005931 | peptide(di) | NA | 5 | -2.22 |
| Ile-Ala | 0.004202 | peptide(di) | NA | 5 | 1.35 |
| Lys-His | 0.000488 | peptide(di) | NA | 7 | -4.16 |
| Phe-Asp | 0.000107 | peptide(di) | NA | 5 | -1.48 |
| Lys-Pro | 0.000107 | peptide(di) | NA | 7 | -1.52 |
| Ile-Phe | 5.57E-05 | peptide(di) | NA | 5 | 1.60 |
| Ser-Arg | 3.26E-05 | peptide(di) | NA | 7 | -2.14 |
| Val-Tyr | 2.45E-05 | peptide(di) | NA | 5 | 1.89 |
| Phe-Thr | 8.31E-06 | peptide(di) | NA | 5 | -6.24 |
| Pro-Arg | 5.1E-06 | peptide(di) | NA | 5 | -2.24 |
| Aspartyl-L-proline | 4.71E-06 | peptide(di) | NA | 5 | -4.21 |
| Trp-Ala | 3.81E-06 | peptide(di) | NA | 5 | -1.78 |
| Trp-Ser | 8.54E-07 | peptide(di) | NA | 5 | -2.60 |
| Asp-Arg | 2.48E-07 | peptide(di) | NA | 7 | -6.98 |
| Asn-Pro | 2.18E-07 | Peptide(di) | NA | 5 | -2.56 |
| Glycylproline | 4.56E-08 | peptide(di) | NA | 5 | -1.56 |
| Thr-Tyr | 2.89E-08 | peptide(di) | NA | 5 | -2.06 |
| Lys-Tyr | 8.7E-10 | Peptide(di) | NA | 5 | -6.64 |
| Arg-His | 3.29E-10 | Peptide(di) | NA | 5 | -6.28 |
| Glu-Glu | 0.000202 | peptide(di) | C01425 | 5 | -4.30 |
| L-Tyrosyl-L-arginine | 1.81E-07 | peptide(di) | C02993 | 7 | -3.62 |
| His-Leu | 0.003736 | peptide(di) | C05010 | 7 | 1.01 |
| Ala-Leu-Val-Ser | 0.024488 | peptide(tetra) | NA | 5 | -2.09 |
| Ile-Ile-Pro-Ser | 0.020917 | peptide(tetra) | NA | 5 | -3.29 |
| Ala-Asp-Gly-His | 0.018444 | peptide(tetra) | NA | 5 | -2.83 |
| Ala-Leu-Pro-Ser | 0.017672 | peptide(tetra) | NA | 5 | -1.26 |
| Pro-Val-Val-Pro | 0.009605 | peptide(tetra) | NA | 5 | -2.80 |
| Arg-Leu-Thr-Ser | 0.009486 | peptide(tetra) | NA | 7 | -2.15 |
| Asn-Trp-Trp-Cys | 0.009047 | peptide(tetra) | NA | 5 | -3.59 |
| Ala-Leu-Ala-Gln | 0.007403 | peptide(tetra) | NA | 5 | -3.29 |
| Ala-Lys-Gln-Pro | 0.005351 | peptide(tetra) | NA | 7 | -1.51 |
| Ala-Glu-Ile-Val | 0.004531 | peptide(tetra) | NA | 5 | -3.04 |
| Asn-Leu-Val-Pro | 0.004333 | peptide(tetra) | NA | 7 | -1.98 |
| Ala-Ala-Pro-Arg | 0.002297 | peptide(tetra) | NA | 5 | -4.47 |
| Ala-Phe-Gln-Pro | 0.00217 | peptide(tetra) | NA | 5 | -3.95 |
| Arg-Lys-Gly-His | 0.00214 | peptide(tetra) | NA | 5 | -9.44 |
| Asp-Glu-Ile-Trp | 0.001983 | peptide(tetra) | NA | 5 | -4.27 |
| Ala-Ala-Ala-Pro | 0.001404 | peptide(tetra) | NA | 5 | -4.12 |
| Asp-Leu-Pro-Ser | 0.001167 | peptide(tetra) | NA | 5 | -3.99 |
| Arg-Trp-Trp-Gly | 0.001014 | peptide(tetra) | NA | 5 | -4.67 |
| Ile-Val-Pro-Ser | 0.00101 | peptide(tetra) | NA | 7 | -1.05 |
| Ile-Ile-Ile-Pro | 0.000916 | peptide(tetra) | NA | 5 | -2.39 |
| Ala-Pro-Ser-Ser | 0.000844 | peptide(tetra) | NA | 5 | -3.44 |
| Asp-Met-Asp-Gly | 0.000776 | peptide(tetra) | NA | 5 | 1.18 |
| Asn-Thr-Gln-Tyr | 0.00071 | peptide(tetra) | NA | 5 | -2.80 |
| Ala-Met-Gln-Tyr | 0.000551 | peptide(tetra) | NA | 5 | -3.52 |
| Pro-Val-Pro-Ser | 0.000482 | peptide(tetra) | NA | 7 | -5.81 |
| Ala-Lys-Cys-His | 0.000481 | peptide(tetra) | NA | 5 | -1.14 |
| Ala-Phe-Gly-Arg | 0.000283 | peptide(tetra) | NA | 5 | -6.52 |
| Arg-Val-Gly-Pro | 0.000276 | peptide(tetra) | NA | 5 | -6.74 |
| Ala-Phe-Asp-Arg | 0.000266 | peptide(tetra) | NA | 5 | 3.83 |
| Ala-Asp-Pro-Pro | 0.000174 | peptide(tetra) | NA | 5 | -6.87 |
| Asn-Leu-Phe-Asp | 0.000102 | peptide(tetra) | NA | 5 | -7.62 |
| Asp-Ile-Ile-Pro | 0.000066 | peptide(tetra) | NA | 5 | -2.25 |
| Arg-Cys-Pro-Arg | 6.09E-05 | peptide(tetra) | NA | 5 | -4.42 |
| Ala-Met-Gly-Ser | 5.13E-05 | peptide(tetra) | NA | 7 | -7.28 |
| Ala-Gly-Pro-His | 0.000038 | peptide(tetra) | NA | 5 | -5.61 |
| Ala-Asp-Asp-Gln | 3.41E-05 | peptide(tetra) | NA | 5 | -6.90 |
| Asn-Pro-Pro-His | 2.08E-05 | peptide(tetra) | NA | 5 | -4.01 |
| Asn-Lys-Gly-Pro | 1.23E-05 | peptide(tetra) | NA | 5 | -2.18 |
| His-Lys-Phe-His | 1.12E-05 | peptide(tetra) | NA | 5 | -4.12 |
| Glu-Phe-Pro-Pro | 1.02E-05 | peptide(tetra) | NA | 5 | -5.39 |
| Ala-Gly-Pro-Pro | 9.3E-06 | peptide(tetra) | NA | 5 | -6.83 |
| Gln-Phe-Pro-His | 4.84E-06 | peptide(tetra) | NA | 7 | 1.51 |
| Ala-Leu-Thr-Pro | 3.5E-06 | peptide(tetra) | NA | 5 | -2.08 |
| Lys-Thr-Ser-Tyr | 2.04E-06 | peptide(tetra) | NA | 7 | -9.60 |
| Gln-Phe-Pro-Pro | 1.18E-06 | peptide(tetra) | NA | 5 | -1.76 |
| Asn-Leu-Thr-Pro | 6.77E-07 | peptide(tetra) | NA | 5 | -1.80 |
| Arg-Gly-Ser-Tyr | 4.2E-07 | peptide(tetra) | NA | 5 | -4.82 |
| Arg-Cys-Gly-Arg | 2.99E-07 | peptide(tetra) | NA | 5 | -1.68 |
| Asp-Leu-Thr-Ser | 2.71E-08 | peptide(tetra) | NA | 7 | -4.57 |
| Gln-Leu-Lys-Pro | 2.57E-08 | peptide(tetra) | NA | 5 | -3.59 |
| Ala-Val-Gly-Pro | 1.98E-08 | peptide(tetra) | NA | 5 | -3.49 |
| Cys-Leu-Met-Trp | 1.75E-08 | peptide(tetra) | NA | 5 | -2.97 |
| Arg-Thr-Ser-Tyr | 1.35E-08 | peptide(tetra) | NA | 5 | -3.05 |
| Ile-Ile-Pro-Val | 1.28E-08 | peptide(tetra) | NA | 5 | -2.93 |
| Ala-Leu-Gly-Pro | 1.02E-08 | peptide(tetra) | NA | 5 | -3.45 |
| Gln-Pro-His-His | 3.34E-09 | peptide(tetra) | NA | 7 | -3.55 |
| Glu-Glu-Gln-Phe | 2.01E-09 | peptide(tetra) | NA | 5 | -3.22 |
| Phe-Pro-Pro-Tyr | 1.31E-09 | peptide(tetra) | NA | 5 | -2.15 |
| Ile-Lys-Thr-Tyr | 6.26E-10 | peptide(tetra) | NA | 7 | -5.84 |
| Ala-Leu-Trp-Asp | 4.62E-10 | peptide(tetra) | NA | 5 | -4.31 |
| Asp-Ile-Ile-Trp | 4.19E-10 | peptide(tetra) | NA | 5 | -5.35 |
| Asn-Leu-Pro-Pro | 2.62E-10 | peptide(tetra) | NA | 7 | -5.98 |
| Ala-Glu-Glu-Pro | 1.3E-10 | peptide(tetra) | NA | 5 | -3.16 |
| Ile-Val-Pro-Pro | 2.53E-11 | peptide(tetra) | NA | 5 | -8.27 |
| Ile-Phe-Pro-Pro | 1.26E-11 | peptide(tetra) | NA | 7 | -4.33 |
| Asp-Met-Met-Tyr | 8.21E-12 | peptide(tetra) | NA | 7 | -7.29 |
| Ala-Leu-Trp-Val | 6.78E-12 | peptide(tetra) | NA | 7 | -8.04 |
| Ala-Leu-Ala-Ser | 4.08E-12 | peptide(tetra) | NA | 5 | -6.84 |
| Asp-Leu-Pro-Pro | 2.66E-12 | peptide(tetra) | NA | 7 | -4.65 |
| Lys-Ser-Ser-Tyr | 1.12E-12 | peptide(tetra) | NA | 5 | -8.16 |
| Glu-Ile-Ile-Pro | 6.59E-13 | peptide(tetra) | NA | 5 | -7.53 |
| Asp-Trp-Gly-Gly | 1.38E-13 | peptide(tetra) | NA | 5 | -10.53 |
| Ala-Ala-Pro-Pro | 1.26E-13 | peptide(tetra) | NA | 5 | -8.85 |
| Ala-Thr-Thr-Ser | 4.73E-14 | peptide(tetra) | NA | 5 | -7.71 |
| Arg-Phe-Gln-Pro | 4.62E-14 | peptide(tetra) | NA | 5 | -6.58 |
| Gln-Leu-Pro-Pro | 3.87E-14 | peptide(tetra) | NA | 5 | -8.02 |
| Gln-Met-Phe-Tyr | 2.48E-14 | peptide(tetra) | NA | 5 | -8.09 |
| Glu-Ala-Val | 1.5E-14 | peptide(tetra) | NA | 7 | -8.58 |
| Ala-Pro-Ser-Arg | 1.36E-14 | peptide(tetra) | NA | 5 | -6.69 |
| Ala-Ala-Asp-Pro | 9.72E-15 | peptide(tetra) | NA | 5 | -7.91 |
| Arg-Met-Cys-Pro | 9.72E-15 | peptide(tetra) | NA | 5 | -7.35 |
| Ala-Val-Pro-Pro | 3.81E-15 | peptide(tetra) | NA | 5 | -9.07 |
| Ala-Asn-Cys-Gly | 2.44E-15 | peptide(tetra) | NA | 5 | -6.26 |
| Asp-Leu-Trp-Pro | 2.26E-15 | peptide(tetra) | NA | 5 | -7.93 |
| Lys-Thr-Thr-Thr | 1.64E-15 | peptide(tetra) | NA | 5 | -7.63 |
| Arg-Met-Trp-Trp | 1.44E-15 | peptide(tetra) | NA | 5 | -7.51 |
| Arg-Trp-Gln-Pro | 1.12E-15 | peptide(tetra) | NA | 5 | -9.87 |
| Glu-Ile-Gln-Pro | 6.68E-16 | peptide(tetra) | NA | 5 | -6.83 |
| Asn-Asp-Gly-Pro | 6.59E-16 | peptide(tetra) | NA | 5 | -8.41 |
| Ala-Leu-Ser-His | 5.28E-16 | peptide(tetra) | NA | 5 | -8.63 |
| Ala-Ser-Arg-Arg | 4.93E-16 | peptide(tetra) | NA | 7 | -7.61 |
| Ala-Lys-Gly-Gly | 3.86E-16 | peptide(tetra) | NA | 7 | 6.72 |
| Ala-Ile-Ile-Pro | 3.18E-16 | peptide(tetra) | NA | 5 | -10.17 |
| Ala-Lys-Phe-Gly | 2.18E-16 | peptide(tetra) | NA | 5 | -7.42 |
| Ala-Cys-Arg-His | 5.65E-17 | peptide(tetra) | NA | 5 | -6.11 |
| His-Lys-Met-His | 3.39E-17 | peptide(tetra) | NA | 5 | -7.76 |
| Arg-Asn-Gly-Tyr | 1.6E-17 | peptide(tetra) | NA | 5 | -7.72 |
| His-Gly-Pro-Pro | 1.53E-17 | peptide(tetra) | NA | 5 | -8.39 |
| Asp-Glu-Ile-Met | 1.21E-17 | peptide(tetra) | NA | 5 | -7.40 |
| Asp-Leu-Trp-Val | 1.45E-18 | peptide(tetra) | NA | 5 | -9.74 |
| Asn-Met-Asn-Cys | 2.47E-20 | peptide(tetra) | NA | 5 | -9.38 |
| Ala-Gly-Pro-Tyr | 7.26E-24 | peptide(tetra) | NA | 5 | -9.70 |
| Ile-Ala-Gly | 0.030008 | Peptide(tri) | NA | 7 | 2.94 |
| Ile-Ser-Val | 0.018993 | Peptide(tri) | NA | 5 | -1.00 |
| Glu-Ala-Phe | 0.008934 | Peptide(tri) | NA | 7 | -2.36 |
| Val-Pro-His | 0.007657 | Peptide(tri) | NA | 5 | -3.23 |
| Pro-Ser-Arg | 0.004205 | Peptide(tri) | NA | 5 | -3.38 |
| Phe-Ala-Arg | 0.003574 | Peptide(tri) | NA | 5 | -5.15 |
| Ile-Lys-Tyr | 0.003347 | Peptide(tri) | NA | 5 | -4.09 |
| Val-Pro-Pro | 0.00124 | Peptide(tri) | NA | 5 | -3.92 |
| Val-Val-His | 0.000951 | Peptide(tri) | NA | 5 | -3.33 |
| Asn-Gly-Pro | 0.000741 | Peptide(tri) | NA | 5 | -4.04 |
| Ala-Asp-Pro | 0.0006 | Peptide(tri) | NA | 5 | -4.99 |
| Lys-Val-Ser | 0.000466 | Peptide(tri) | NA | 5 | -3.52 |
| Lys-Val-Pro | 0.000396 | Peptide(tri) | NA | 5 | -3.60 |
| Ile-Ile-Met | 0.000148 | Peptide(tri) | NA | 7 | -6.83 |
| Val-Val-Pro | 0.000145 | Peptide(tri) | NA | 5 | -2.10 |
| Glu-Ala-Thr | 0.000132 | Peptide(tri) | NA | 5 | -6.39 |
| Ile-Arg-Pro | 0.000129 | Peptide(tri) | NA | 5 | -4.58 |
| Ala-Gly-Ser | 0.000112 | Peptide(tri) | NA | 7 | -1.54 |
| Ala-Ala-Pro | 0.000105 | Peptide(tri) | NA | 7 | -1.57 |
| Lys-Ala-Pro | 7.62E-05 | Peptide(tri) | NA | 5 | -5.23 |
| Glu-Ile-Pro | 2.69E-05 | Peptide(tri) | NA | 5 | -1.18 |
| Lys-Val-His | 0.000025 | Peptide(tri) | NA | 5 | -3.39 |
| Ile-Lys-Ser | 5.55E-06 | Peptide(tri) | NA | 5 | -1.52 |
| Ile-Lys-Pro | 9.97E-07 | Peptide(tri) | NA | 5 | -2.11 |
| Asn-Asn-Asn | 3.74E-07 | Peptide(tri) | NA | 5 | -1.96 |
| Ala-Pro-Ser | 3.69E-07 | Peptide(tri) | NA | 5 | -6.12 |
| Glu-Ser-Val | 1.73E-07 | Peptide(tri) | NA | 5 | -2.74 |
| Lys-Trp-Pro | 9.65E-08 | Peptide(tri) | NA | 5 | -1.26 |
| Ile-Pro-Ser | 1.84E-08 | peptide(tri) | NA | 5 | -2.27 |
| Glu-Pro-Tyr | 4.43E-09 | peptide(tri) | NA | 5 | -2.43 |
| Ile-Pro-Thr | 1.31E-09 | peptide(tri) | NA | 5 | -3.86 |
| Phe-Pro-Pro | 9.1E-10 | peptide(tri) | NA | 5 | -4.16 |
| Ile-Pro-Pro | 5.03E-10 | peptide(tri) | NA | 5 | -3.28 |
| Ala-Gly-Pro | 1.13E-10 | peptide(tri) | NA | 7 | -5.29 |
| Phe-Phe-Pro | 1.41E-13 | peptide(tri) | NA | 5 | -9.66 |
| Glu-Asp-His | 4.82E-14 | peptide(tri) | NA | 5 | -9.19 |
| Glu-Glu-Ile | 1.22E-14 | peptide(tri) | NA | 5 | -6.20 |
| Asp-Pro-Ser | 5.72E-15 | peptide(tri) | NA | 5 | -8.85 |
| Glu-Pro-Thr | 2.64E-15 | peptide(tri) | NA | 7 | -7.50 |
| Glu-Pro-Ser | 2.23E-15 | peptide(tri) | NA | 5 | -8.27 |
| Lys-Val-Tyr | 1.77E-15 | peptide(tri) | NA | 5 | -7.65 |
| Lys-Pro-Pro | 1.16E-16 | peptide(tri) | NA | 5 | -9.51 |
| Glu-Phe-Pro | 1.27E-17 | peptide(tri) | NA | 5 | -6.69 |
| Glu-Pro-Pro | 9.06E-18 | peptide(tri) | NA | 5 | -9.59 |
| Glu-Ala-Ser | 3.53E-18 | peptide(tri) | NA | 5 | -6.90 |
| Ala-Pro-Pro | 1.44E-18 | peptide(tri) | NA | 5 | -7.62 |
| Gly-Pro-Pro | 4.06E-19 | peptide(tri) | NA | 7 | -8.32 |
| Lys-Phe-Pro | 1.36E-19 | peptide(tri) | NA | 5 | -9.48 |
| Leu-Gly-Pro | 9.32E-09 | peptide(tri) | C01833 | 7 | -2.52 |
| N3-(4-methoxyfumaroyl)-L-2,3-diaminopropanoate | 0.022486 | Undefined | NA | 7 | 2.26 |
| L-alpha-glutamyl-L-hydroxyproline | 0.00815 | Undefined | NA | 5 | -3.55 |
| N-Decanoylglycine | 0.004839 | Undefined | NA | 5 | 1.02 |
| 10,11-dihydro-20-trihydroxy-leukotriene B4 | 0.003743 | Undefined | NA | 7 | -1.08 |
| 6-Dimethylaminopurine | 0.001272 | Undefined | NA | 5 | -3.57 |
| Epsilon-(gamma-Glutamyl)-lysine | 0.000298 | Undefined | NA | 5 | -1.31 |
| 1,6-anhydro-N-acetylmuramate | 0.000101 | Undefined | NA | 8 | -2.06 |
| Glycylprolylhydroxyproline | 9.84E-05 | Undefined | NA | 5 | -6.15 |
| 3'-amino-3'-deoxyadenosine | 9.66E-05 | Undefined | NA | 7 | -7.11 |
| 1-deoxyxylonojirimycin | 3.66E-05 | Undefined | NA | 7 | -3.49 |
| 5-Methoxytryptophan | 3.12E-05 | Undefined | NA | 5 | -1.77 |
| Succinyladenosine | 1.83E-05 | Undefined | NA | 5 | -6.49 |
| 2'-methoxythiamin pyrophosphate | 1.62E-05 | Undefined | NA | 7 | 6.95 |
| 3-Nitrotyrosine | 7.11E-06 | Undefined | NA | 5 | -6.55 |
| L-thiazolidine-4-carboxylate | 4.14E-06 | Undefined | NA | 5 | -4.18 |
| Vinylacetylglycine | 1.17E-06 | Undefined | NA | 5 | -1.18 |
| glcNAc-1,6-anhMurNAc | 5.47E-07 | Undefined | NA | 8 | -1.91 |
| L-isoleucyl-L-proline | 1.36E-07 | Undefined | NA | 5 | -2.83 |
| N-Acetylcadaverine | 5.07E-08 | Undefined | NA | 7 | -6.04 |
| 8-Hydroxy-7-methylguanine | 4.22E-08 | Undefined | NA | 5 | -2.32 |
| N-Acetylglutamine | 2.94E-08 | Undefined | NA | 5 | -2.83 |
| dihydrothymidine | 2.02E-08 | Undefined | NA | 5 | -3.21 |
| N1,N8-diacetylspermidine | 1.55E-08 | Undefined | NA | 5 | -3.49 |
| tetrahydropteroate | 8.24E-09 | Undefined | NA | 5 | -2.54 |
| N-acetyl -D- glucosaminitol | 2.86E-09 | Undefined | NA | 5 | -3.66 |
| &gamma;-thiomethyl glutamate | 2.77E-10 | Undefined | NA | 5 | -4.29 |
| N-acetyl-(L)-arginine | 1.7E-11 | Undefined | NA | 7 | -7.19 |
| 6-deoxy-5-ketofructose-1-phosphate | 1.44E-12 | Undefined | NA | 6 | -6.39 |
| 2-oxobut-3-enanoate | 8.95E-13 | Undefined | NA | 5 | -4.96 |
| 2,3-dichloro-5-methyl-muconate | 5.51E-14 | Undefined | NA | 5 | -9.83 |
| L-methioninamide | 2.87E-14 | Undefined | NA | 5 | -7.99 |
| 5-Methylcytidine | 1.16E-14 | Undefined | NA | 7 | -6.91 |
| P-DPD | 8.17E-15 | Undefined | NA | 5 | -7.05 |
| 2-hydroxy-4-methylthiobutanoate | 3.52E-17 | Undefined | NA | 7 | -11.02 |
| phenylhydantoin | 9.98E-18 | Undefined | NA | 8 | -10.40 |
| Dihydrozeatin-9-N-glucoside-O-glucoside | 2.59E-18 | Undefined | NA | 5 | -6.90 |
| ADP | 1.5E-15 | Undefined | C0008 | 6 | -9.45 |
| Palmitoylglycerone phosphate | 5.29E-05 | Undefined | C01192 | 5 | -1.56 |
| Methyl 2-diazoacetamidohexonate | 0.000469 | Undefined | C01223 | 5 | -1.58 |
| N-6-Aminohexanoyl-6-aminohexanoate | 0.000101 | Undefined | C01255 | 7 | 1.32 |
| N-(6-Aminohexanoyl)-6-aminohexanoate | 1.83E-06 | Undefined | C01255 | 7 | -2.46 |
| Arg-OEt | 3.21E-05 | Undefined | C01404 | 5 | -5.10 |
| 2-Furoate | 5.24E-15 | Undefined | C01546 | 5 | -8.99 |
| D-Ornaline | 8.08E-06 | Undefined | C01683 | 5 | -4.03 |
| Nonanoyl-CoA | 0.000441 | Undefined | C01942 | 7 | -4.06 |
| Retinyl ester | 0.006443 | Undefined | C02075 | 5 | 1.36 |
| 3-Methylguanine | 1.94E-10 | Undefined | C02230 | 6 | -5.92 |
| Phosphoramidate | 0.001095 | Undefined | C02306 | 5 | 2.21 |
| N-Ethylmaleimide | 2.32E-05 | Undefined | C02441 | 5 | -1.60 |
| 1-Methyladenosine | 2.53E-14 | Undefined | C02494 | 5 | -7.13 |
| Tolylacetonitrile | 0.0049 | Undefined | C02596 | 5 | -3.44 |
| N-Acetyl-L-leucine | 0.008105 | Undefined | C02710 | 7 | -5.63 |
| N-Acetylmethionine | 0.023634 | Undefined | C02712 | 5 | -3.18 |
| N-Acetylmuramate | 1.88E-08 | Undefined | C02713 | 8 | -2.60 |
| 8-Oxodeoxycoformycin | 1.77E-07 | Undefined | C02957 | 7 | -2.20 |
| N-Acetyl-L-histidine | 1.3E-10 | Undefined | C02997 | 7 | -6.67 |
| 3-Hydroxyhexobarbital | 6E-16 | Undefined | C03068 | 5 | -6.87 |
| Diisopropyl phosphate | 2.89E-07 | Undefined | C03113 | 5 | -1.95 |
| Pyrimidine nucleoside | 7.34E-07 | Undefined | C03169 | 5 | -1.82 |
| D-2-Hydroxyisocaproate | 6.39E-08 | Undefined | C03264 | 5 | -7.92 |
| Glycerophosphoglycerol | 7.97E-12 | Undefined | C03274 | 7 | -7.64 |
| 2-Ethylhexyl phthalate | 0.000347 | Undefined | C03343 | 5 | 1.19 |
| N6,N6-Dimethyladenosine | 3.96E-07 | Undefined | C03416 | 5 | -1.70 |
| Bis(2-ethylhexyl)phthalate | 0.028902 | Undefined | C03690 | 5 | 1.14 |
| S-Acetylphosphopantetheine | 0.005035 | Undefined | C03725 | 5 | -2.39 |
| N-Hydroxy-2-acetamidofluorene | 0.005263 | Undefined | C03954 | 5 | -2.88 |
| 4-(Trimethylammonio)but-2-enoate | 2.25E-07 | Undefined | C04114 | 7 | -3.15 |
| 2,7-Anhydro-alpha-N-acetylneuraminic acid | 1.82E-10 | Undefined | C04521 | 5 | -5.74 |
| gamma-L-Glutamyl-L-cysteinyl-beta-alanine | 1.55E-13 | Undefined | C04544 | 7 | -7.93 |
| Isoquinoline | 0.00121 | Undefined | C06323 | 6 | -1.44 |
| 5'-Butyrylphosphouridine | 1.52E-15 | Undefined | C06436 | 5 | 8.11 |
| Muramic acid | 1.92E-08 | Undefined | C06470 | 5 | -2.15 |
| cis-1,2-Dihydroxy-1,2-dihydrodibenzothiophene | 0.000259 | Undefined | C06721 | 5 | -1.17 |
| 2-O-alpha-L-Rhamnopyranosyl-D-glucopyranose | 9.41E-15 | Undefined | C08244 | 5 | -7.39 |
| L-gamma-Glutamyl-L-hypoglycin | 0.0073 | Undefined | C08280 | 6 | -4.16 |
| Ichangin | 5.39E-07 | Undefined | C08768 | 5 | -2.74 |
| 8-(3,3-Dimethylallyl)spatheliachromene | 1.88E-06 | Undefined | C09002 | 5 | -1.52 |
| Trilobolide | 5.9E-07 | Undefined | C09563 | 5 | -4.84 |
| alpha-Irone | 0.002703 | Undefined | C09690 | 7 | 1.58 |
| Deoxymannojirimycin | 3.31E-11 | Undefined | C10141 | 7 | -5.58 |
| Danielone | 9.19E-06 | Undefined | C10674 | 5 | -3.62 |
| Bicozamycin | 3.25E-06 | Undefined | C11259 | 5 | -2.08 |
| Leucyl-leucyl-norleucine | 9.15E-07 | Undefined | C11328 | 5 | -1.31 |
| N-Acetyl-leucyl-leucine | 0.0005 | Undefined | C11333 | 7 | -4.68 |
| 5'-Dehydroadenosine | 1.14E-05 | Undefined | C11500 | 5 | -3.50 |
| Succinyl proline | 0.00156 | Undefined | C11711 | 5 | -5.56 |
| Cyclic ADP-ribose | 5.18E-10 | Undefined | C13050 | 5 | -6.66 |
| (S)-ATPA | 2.54E-05 | Undefined | C13733 | 7 | -1.17 |
| 1-Hexadecanoyl-2-(9Z-octadecenoyl)-sn-glycero-3-phospho-sn-glycerol 3'-phosphate | 0.000122 | Undefined | C13885 | 5 | -1.76 |
| Di(2-ethylhexyl) adipate | 0.013884 | Undefined | C14240 | 5 | 2.39 |
| Tributyl phosphate | 0.016219 | Undefined | C14439 | 5 | 1.24 |
| (R)-4'-Deoxyindenestrol | 3.63E-06 | Undefined | C14541 | 7 | -4.31 |
| 1-(beta-D-Ribofuranosyl)-1,4-dihydronicotinamide | 1.59E-07 | Undefined | C15497 | 8 | -8.13 |
| Mugineic acid | 2.14E-05 | Undefined | C15500 | 5 | -6.53 |
| Mycinamicin VI | 0.000473 | Undefined | C15682 | 5 | -6.26 |
| L-Pyrrolysine | 0.002363 | Undefined | C16318 | 5 | -2.55 |
| dihomocitrate | 6.78E-16 | Undefined | C16583 | 5 | -8.09 |
| allylcysteine | 7.34E-19 | Undefined | C16759 | 5 | -9.20 |

**Supplementary Table S3.** Primers employed for qPCR analysis.

| **Name of primer** | **Sequence (5′ to 3′)** | **Amplicon length** |
| --- | --- | --- |
| **ompC forward** | CAGGCGAACAACACTGAAAG | 102 |
| **ompC reverse** | GTTACGACCGTAGTCGAAAGAG | 102 |
| **ribC forward** | GAAAGAGACGCTGCGGATTA | 97 |
| **ribC reverse** | CCCGCCGATTTCATCACTAA | 97 |
| **nfsB forward** | ATCAGTGGATGGCGAAACAG | 138 |
| **nfsB reverse** | CCTGGGCTTTCAGGTCAAAT | 138 |
| **oqxB forward** | GGTATCGCTAACCGAGCTATTC | 83 |
| **oqxB reverse** | TACGCCCGAACTGGTTAAAG | 83 |
| **acrA forward** | CGCTAACAGGATGTGACGATAA | 92 |
| **acrA reverse** | TTGTAGAGGTGCGGATTTGAG | 92 |
| **acrB forward** | GGTCGTTATCTGGTGCTGTATC | 117 |
| **acrB reverse** | CTGCGCCATACTCAGGAATAC | 117 |

**Supplementary Text**

**Impacted** **metabolites in central carbohydrate metabolism in *K. pneumoniae* INF348 induced by nitrofurantoin treatment**

The TCA cycle is pivotal biological process that regulates bacterial cellular respiration and provides various key energy precursors, such as succinate and citrate, which are needed for fatty acids biosynthesis.^2^ At 1 and 4 h, NFT treatment produced a significant suppression in the levels of three intermediates involved in the glycolysis pathway, namely 3-phospho-D-glycerate, phosphoenolpyruvate and pyruvate (log_2_FC ≥ -1.0, *p* ≤ 0.05; **Supplementary Figure S6A& B**). Consequently, the TCA cycle (directly downstream) was also significantly perturbed, following NFT treatment at 1 h the abundance of ten key precursors significantly depleted, specifically acetyl-CoA, pyruvate, 2-oxoglutarate, succinyl-CoA, succinate, phosphoenolpyruvate, 3-phospho-D-glycerate, (*S*)-malate, GTP and CoA (log_2_FC ≥ -3.0, *p* ≤ 0.05) (**Supplementary Figure S6A& B**). However, a greater degree of metabolite suppression was evident at 4 h, which manifested as a marked reduction in the levels of 12 fundamental TCA elements, namely 2-(alpha-hydroxyethyl)thiamine diphosphate, acetyl-CoA, pyruvate, 3-phospho-D-glycerate, phosphoenolpyruvate, citrate, 2-oxoglutarate, succinyl-CoA, succinate, (*S*)-malate, GTP and CoA (log_2_FC= -2.5, -4.5, -1.8, -5.3, -3.7, -1.7, -7.7, -7.7, -6.9, -4.3, -5.1 and -6.0, respectively; **Supplementary Figure S6A**).

**Impacted metabolites in pantothenate and CoA biosynthesis in *K. pneumoniae* INF348 induced by nitrofurantoin treatment**

Pantothenate and CoA are essential co-factors for enzymes involved in the biogenesis of phospholipids and fatty acids, and the TCA cycle.^3^ Notably, the levels of ten key precursors of pantothenate and CoA biosynthesis were significantly depleted after NFT treatment at 1 h specifically, pyruvate, (*R*)-pantothenate, (*R*)-pantetheine, 3-methyl-2-oxobutanoic acid, pantetheine 4'-phosphate, L-valine, (*R*)-pantoate, dephospho-CoA, 2-acetolactate and CoA (log_2_FC ≥ -1.0, *p* ≤ 0.05) (**Supplementary Figure S7**). A slightly higher level of overrepresentation was observed in the pantothenate and CoA biosynthesis owing to NFT treatment at 4 h, wherein the abundance of eleven main components experienced a significant depletion, specifically pyruvate, (*R*)-pantothenate, (*R*)-pantetheine, 3-methyl-2-oxobutanoic acid, pantetheine 4'-phosphate, L-valine, (*R*)-2,3-dihydroxy-3-methylbutanoate, (*R*)-pantoate, dephospho-CoA, 2-acetolactate and CoA (log_2_FC ≥ -2.0, *p* ≤ 0.05) (**Supplementary Figure S7**).

**References**

**1.** Creek, D. J.; Jankevics, A.; Burgess, K. E.; Breitling, R.; Barrett, M. P., IDEOM: an Excel interface for analysis of LC-MS-based metabolomics data. *Bioinformatics* **2012,** *28* (7), 1048-9.

**2.** Gest, H. In *Evolutionary roots of the citric acid cycle in prokaryotes*, Biochemical Society Symposium, 1987; pp 3-16.

**3.** Leonardi, R.; Jackowski, S., Biosynthesis of Pantothenic Acid and Coenzyme A. *EcoSal Plus* **2013,** *1* (3), 1.
